# Guselkumab Reduces Disease- and Mechanism-Related Biomarkers More Than Adalimumab in Patients with Psoriasis: A VOYAGE 1 Substudy

**DOI:** 10.1016/j.xjidi.2024.100287

**Published:** 2024-06-05

**Authors:** Andrew Blauvelt, Richard G. Langley, Patrick J. Branigan, Xuejun Liu, Yanqing Chen, Samuel DePrimo, Keying Ma, Brittney Scott, Kim Campbell, Ernesto J. Muñoz-Elías, Kim A. Papp

**Affiliations:** 1 Oregon Medical Research Center, Portland, Oregon, USA; 2 Division of Dermatology, Department of Medicine, Dalhousie University, Halifax, Nova Scotia, Canada; 3 Immunology, Janssen Research & Development, LLC, Spring House, Pennsylvania, USA; 4 Immunology, Janssen Research & Development, LLC, San Diego, California, USA; 5 K. Papp Alliance Clinical Trials and Probity Medical Research, Waterloo, ON, Canada; 6 University of Toronto, Toronto, ON, Canada

**Keywords:** Adalimumab, Biomarkers, Guselkumab, IL-23/IL-17 pathway, Psoriasis

## Abstract

**Background:**

Psoriasis is an immune-mediated inflammatory disease characterized by activation of IL-23–driven IL-17–producing T cell and other IL-23 receptor–positive IL-17–producing cell responses. Selective blockade of IL-23p19 with guselkumab was superior to blockade of TNF-α with adalimumab (ADA) in treating moderate-to-severe psoriasis. Objective: Pharmacodynamic responses of guselkumab versus ADA were compared in patients with psoriasis in VOYAGE 1.

**Design:**

Inflammatory cytokine serum levels were assessed (n = 118), and lesional and nonlesional skin biopsies were collected (n = 38) in patient subsets at baseline and 4, 24, and 48 weeks after treatment to evaluate pharmacodynamic responses of guselkumab versus those of ADA.

**Results:**

Guselkumab provided rapid reductions in serum IL-17A, IL-17F, and IL-22 levels by week 4 versus at baseline, which were maintained through weeks 24 and 48 (*P* < .001). The magnitude of reduction of IL-17A and IL-22 at week 48 and IL-17F at weeks 4, 24, and 48 were greater with guselkumab than with ADA (all *P* < .05). In the skin, guselkumab reduced the expression of IL-23/IL-17 pathway–associated and psoriasis-associated genes.

**Conclusion:**

These data provide extensive characterization of pharmacodynamic anti-inflammatory responses to IL-23p19 and TNF-α inhibition in human blood and tissue over time with FDA-approved doses of guselkumab and ADA. **Trial registration:**ClinicalTrials.govClinicalTrials.gov (NCT02207231).

## Introduction

Psoriasis, an immune-mediated inflammatory disorder of the skin, is characterized by the activation of IL-23–driven IL-17–producing T (T17) cells and other IL-23 receptor–positive IL-17–producing cell responses (Elloso et al, 2012; Hawkes et al, 2018; Kim et al, 2022; Lowes et al, 2008; Teng et al, 2015). Cytokines involved in the IL-23/IL-17 pathway have been identified as key modulators of immune responses associated with the initiation, progression, and maintenance phases of psoriasis (Blauvelt et al, 2015a). IL-17A, IL-17F, and IL-22 are elevated in the sera and/or skin lesions of patients with psoriasis (Kagami et al, 2010; Krueger et al, 2015, 2012; Sofen et al, 2014). In humans, selective IL-23 blockade has demonstrated clinical benefit in psoriasis and psoriatic arthritis (Blauvelt et al, 2020, 2017; Coates et al, 2022; Deodhar et al, 2020; Gordon et al, 2018; Mease et al, 2020; Papp et al, 2017; Reich et al, 2017a, 2017b; Wolk et al, 2006; Zaba et al, 2007). However, the mechanism of action associated with the sustained response and therapeutic longevity of IL-23 blockade has not been completely defined.

IL-23, a key regulatory cytokine produced by activated antigen-presenting cells, such as inflammatory monocytes and dendritic cells in psoriatic skin (Mehta et al, 2021), affects the expansion and maintenance of T17 cells (eg, CD4^+^ T helper 17 and IL-17–producing CD8^+^ T cells) through transcriptional control regulated by RORγt (Gaffen et al, 2014; Kim et al, 2022; Teng et al, 2015). T17 cells, which secrete the proinflammatory effector molecules IL-17A and IL-17F, are a significant source of IL-17A in psoriatic skin and play a pivotal role in psoriasis pathogenesis (Elloso et al, 2012; Hawkes et al, 2018; Kim et al, 2022). IL-17A directly regulates keratinocyte-expressed genes involved in innate immune defense, including antimicrobial defensins, S100 family proteins, and lipocalin, as well as a range of C-X-C motif chemokine ligands that regulate neutrophil trafficking (Krueger et al, 2012). IL-17F shares a gene locus with, is coexpressed with, and has a similar protein structure and biologic activity as IL-17A; IL-17F is elevated in the sera and lesional skin (LS) of patients with psoriasis (Blauvelt and Chiricozzi, 2018; Elloso et al, 2012; Johansen et al, 2009; Reich et al, 2021; Soderstrom et al, 2017). IL-23 also affects IL-22 production from both T17 and T helper 22 cells, which have been identified as a source of IL-22 in normal and psoriatic blood and skin (Basu et al, 2012; Benham et al, 2013; Hijnen et al, 2013). IL-22 induces keratinocyte hyperplasia, increases synthesis of S100 proteins, accelerates loss of surface keratinocytes, and helps to eliminate pathogens (Fujita, 2013; Nograles et al, 2008). IL-22 can also have effects divergent from those of IL-17 associated with disruption of normal keratinocyte differentiation in psoriasis (Lowes et al, 2014; Nograles et al, 2008). Thus, both T17 and T helper 22 cells are increased in psoriatic plaques, and the cytokines they produce are critical for inflammatory processes in psoriatic skin.

Guselkumab is a fully human, IgG gamma mAb that inhibits IL-23 by targeting the p19 subunit (Sofen et al, 2014). Guselkumab binds selectively to human IL-23 with high specificity and affinity and blocks extracellular IL-23 binding to the cell surface IL-23 receptor, disrupting IL-23–mediated signaling, activation, and cytokine cascades (Zhuang et al, 2016). In the phase 3 VOYAGE 1 trial, guselkumab demonstrated superior efficacy compared with placebo and the TNF-α antagonist adalimumab (ADA) in patients with moderate-to-severe plaque psoriasis (Blauvelt et al, 2017). In this study, peripheral blood and skin samples were collected from patients in VOYAGE 1, and pharmacodynamic (PD) responses to guselkumab (vs ADA) treatment were assessed over 48 weeks.

## Results

### Study population and demographics

As reported previously for the VOYAGE 1 active-comparator study, 837 patients with longstanding moderate-to-severe psoriasis were randomized to placebo (n = 174), guselkumab (n = 329), or ADA (n = 334) and were treated through week 48 (Blauvelt et al, 2017, NCT02207231). Placebo-treated patients crossed over to receive guselkumab from weeks 16 to 48. The overall VOYAGE 1 population was largely male (72.6%) and White (81.7%), with a 15.0-year median disease duration (25th–75th percentiles = 8.9–25.0), median body surface area involvement of 22.0% (25th–75th percentiles = 15.0–35.0), and median PASI of 19.0 (25th–75th percentiles = 15.6–25.5) at study onset, and 74.6 and 25.1% of the patients had a baseline Investigator’s Global Assessment score of 3 and 4, respectively.

Serum samples were collected from all patients, and a random subset of 118 demographically matched patients was identified for serum biomarkers analysis. In addition, LS and non-LS (NL) samples were collected from 38 patients who provided consent to participate in an optional skin biopsy substudy to evaluate PD effects on psoriasis transcriptomics. Baseline demographic and disease characteristics were generally comparable across the randomized treatment groups, although minor imbalances were noted in the skin biopsy population (younger mean age in the ADA group, higher body mass index for the guselkumab group, lower body surface area with psoriasis for the placebo group), which may reflect the overall limited number of participants in this substudy (Table 1).

**Table 1:** Summary of Baseline Demographics and Clinical Disease Characteristics; Participants Randomized at Week 0 in the Serum Biomarker and Skin Biopsy Subpopulations

Characteristic	VOYAGE-1 Biomarker Subpopulation				VOYAGE-1 Skin Biopsy Subpopulation				VOYAGE-1 Overall^1^
	Placebo	Guselkumab	Adalimumab	Total Biomarker Substudy	Placebo	Guselkumab	Adalimumab	Total skin Biopsy Substudy	
Analysis set: Participants randomized at wk 0 in the serum biomarker and skin biopsy subpopulations	40	40	38	118	8	17	13	38	837
Age (y)									
Mean (SD)	47.9 (12.3)	46.8 (12.7)	46.4 (14.7)	47.0 (13.2)	48.0 (9.9)	51.2 (11.7)	43.5 (15.5)	47.9 (12.9)	43.7 (12.7)
Median	49	48	46.5	48	50	53	44	49.5	44
Q1–Q3	(39.0–56.0)	(36.0–57.0)	(35.0–53.0)	(37.0–55.0)	(42.0–53.0)	(45.0–59.0)	(33.0–51.0)	(39.0–57.0)	(33.0–53.0)
Range	(19–68)	(20–76)	(23–82)	(19–82)	(31–63)	(29–70)	(20–67)	(20–70)	(18–87)
Race									
White	34 (85.0%)	34 (85.0%)	34 (89.5%)	102 (86.4%)	8 (100%)	15 (88.2%)	12 (92.3%)	35 (92.1%)	684 (81.7%)
Black or African American	2 (5.0%)	1 (2.5%)	2 (5.3%)	5 (4.2%)	0	1 (5.9%)	1 (7.7%)	2 (5.3%)	17 (2.0%)
Asian	1 (2.5%)	5 (12.5%)	2 (5.3%)	8 (6.8%)	0	1 (5.9%)	0	1 (2.6%)	121 (14.5%)
American Indian or Alaska Native	0	0	0	0	0	0	0	0	0
Native Hawaiian or other Pacific Islander	1 (2.5%)	0	0	1 (0.8%)	0	0	0	0	3 (0.4%)
Other	2 (5.0%)	0	0	2 (1.7%)	0	0	0	0	10 (1.2%)
Multiple	0	0	0	0	0	0	0	0	2 (0.2%)
Unknown	0	0	0	0	0	0	0	0	0
Not reported	0	0	0	0	0	0	0	0	0
Sex									
Male	29 (72.5%)	27 (67.5%)	26 (68.4%)	82 (69.5%)	6 (75.0%)	14 (82.4%)	9 (69.2%)	29 (76.3%)	608 (72.6%)
Female	11 (27.5%)	13 (32.5%)	12 (31.6%)	36 (30.5%)	2 (25.0%)	3 (17.6%)	4 (30.8%)	9 (23.7%)	229 (27.4%)
BMI (kg/m^2^)									
Mean (SD)	31.0 (6.6)	31.7 (7.1)	31.3 (6.7)	31.3 (6.8)	29.4 (3.2)	32.7 (5.4)	30.3 (6.4)	31.1 (5.5)	29.6 (6.5)
Median	29.3	30.4	30.9	30.2	29.2	32.2	29.1	29.8	28.4
Q1–Q3	(26.1–35.9)	(26.9–34.7)	(24.8–35.0)	(26.1–35.4)	(26.9–29.9)	(28.9–37.6)	(25.9–34.4)	(27.0–34.4)	(25.2–33.2)
Range	(20–48)	(21–50)	(22–51)	(20–51)	(26–36)	(25–41)	(22–44)	(22–44)	(16–65)
BSA (%)									
Mean (SD)	20.6 (14.4)	23.2 (17.2)	22.4 (13.5)	22.1 (15.1)	17.8 (8.8)	25.2 (16.1)	22.2 (13.2)	22.6 (13.8)	27.9 (16.7)
Median	15	17	17.5	16	14	24	18	18	22
Q1–Q3	(12.0–22.5)	(14.0–23.0)	(13.0–26.0)	(13.0–24.0)	(12.5–20.0)	(15.0–30.0)	(13.0–31.0)	(13.0–30.0)	(15.0–36.0)
Range	(10–70)	(11–80)	(10–63)	(10–80)	(12–37)	(12–80)	(10–55)	(10–80)	(10–90)
PASI score (0-72)									
Mean (SD)	18.8 (9.4)	18.5 (7.0)	18.8 (7.2)	18.7 (7.9)	17.4 (6.0)	18.0 (7.4)	18.0 (7.4)	17.8 (6.9)	21.9 (9.2)
Median	15.7	15.9	16.4	16	15.3	14.4	15	14.9	19
Q1–Q3	(13.9–19.6)	(14.6–20.3)	(14.4–19.8)	(14.3–19.8)	(12.7–20.8)	(13.6–17.5)	(12.8–18.6)	(12.8–20.3)	(15.4–25.4)
Range	(12–61)	(12–45)	(12–41)	(12–61)	(13–29)	(12–37)	(13–38)	(12–38)	(7–68)

Abbreviations: BMI, body mass index; BSA, body surface area; PASI, psoriasis area and severity index.

Shown are baseline demographic and disease characteristics of patients selected for the serum biomarker substudy (n = 118), skin biopsy study (n = 39), and all 837 patients in VOYAGE-1.

1 Participants randomized at week 0. Shown are baseline demographic and disease characteristics of patients selected for the serum biomarker substudy (n = 118), skin biopsy study (n = 39), and all 837 patients in VOYAGE-1.

### Outcomes

#### PD effect on serum cytokines associated with the IL-23/IL-17 pathway

**Figure 1:**
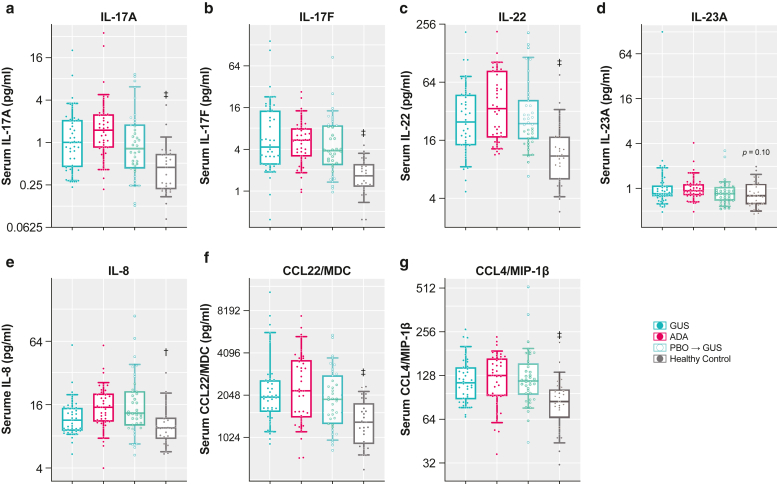
**Serum cytokine levels in psoriasis at baseline versus normal healthy serum control.** (**a**) IL-17A, (**b**) IL-17F, (**c**) IL-22, (**d**) IL-23A, (**e**) IL-8, (**f**) CCL22/MDC, and (**g**) CCL4/MIP-1β. Data are presented as concentration (pg/ml, y-axis in log scale) of a subset of the VOYAGE 1 study population (n = 118) compared with that of serum samples obtained from an independent demographically matched healthy control cohort (n = 25). Boxplots show median (25th–75th percentile) and 10th–90th percentiles (whiskers). *P*-values were derived from Welch’s *t*-test comparing all biomarker substudy patients with psoriasis with healthy controls; adjusted *P*-values were calculated using the Benjamini–Hochberg procedure for correction of multiple comparisons (^†^*P* < .01 and ^‡^*P* < .001). ADA, adalimumab; GUS, guselkumab; MDC, macrophage-derived chemokine; PBO, placebo.

Serum analytes included in this study were selected on the basis of an analysis of previous guselkumab phases 1 and 2 psoriasis studies (Gordon et al, 2015; Sofen et al, 2014) and a literature review (Blauvelt et al, 2015b; Suárez-Fariñas et al, 2012; Teng et al, 2015). Baseline substudy serum samples from patients with psoriasis (n = 118) contained significantly (all *P* < .01) higher concentrations of IL-17A, IL-17F, IL-22, IL-8, CCL22/macrophage-derived chemokine (MDC), and CCL4/MIP-1β than an independent, healthy control serum cohort (n = 25) (Figure 1 and Table 2). Baseline concentrations of these key cytokines were comparable across treatment arms (Figure 1 and Table 2). In contrast, baseline serum IL-23 levels were not significantly different between participants with psoriasis and healthy controls (*P* = .10).

**Table 2:** Baseline Serum Cytokines, Measured as Geometric Mean (pg/ml)

	PBO	GUS	ADA	Total Patients with PsO	HC	PsO Versus HC FC (Adjusted *P-*Value)^1^
Serum cytokines						
n	40	40	38	118	25	
IL-17A	0.91	1.07	1.55	1.14	0.41	2.77 (<.001)
IL-17F	4.25	5.75	5.07	4.99	1.59	3.14 (<.001)
IL-22	27.87	26	36.04	29.57	11.29	2.62 (<.001)
IL-23	0.88	1.05	1.02	0.98	0.83	1.18 (.10)
CXCL8/IL-8	15.69	12.03	15.01	14.15	10.14	1.40 (<.010)
CCL22/MDC	1919.21	2132.25	2285.01	2101.87	1295.24	1.62 (<.001)
CCL4/MIP-1β	119.58	117.24	118.54	118.47	81.59	1.45 (<.001)

Abbreviations: ADA, adalimumab; FC, fold change; GUS, guselkumab; HC, healthy control; MDC, macrophage-derived chemokine; PBO, placebo; PsO, psoriasis.

1 *P*-values are derived from Welch’s *t*-test comparing all biomarker substudy patients with PsO with healthy controls; adjusted *P*-values are calculated using the Benjamini–Hochberg procedure for correction of multiple comparisons.

**Figure 2:**
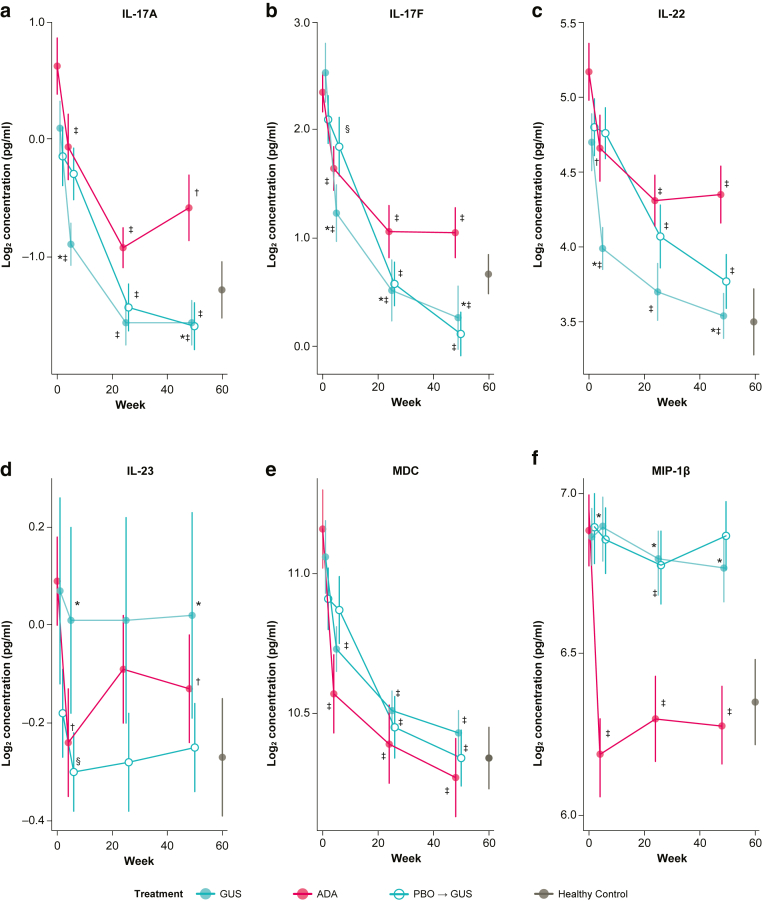
**Selective blockade of IL-23 attenuates IL-17 and IL-22 effector cytokines.** Data are presented as mean log_2_ concentration (pg/ml) over time (plotted means are linear model derived expected LS mean [emmean] plus SE). (**a**) IL-17A, (**b**) IL-17F, (**c**) IL-22, (**d**) IL-23, (**e**) MDC, and (**f**) MIP-1β. PD effects by treatment group at weeks 4, 24, and 48 were evaluated using a paired *t*-test against baseline (^*§*^*P* < .05, ^†^*P* < .01, and ^‡^*P <* .001). Differences in PD effects by GUS (n = 40) versus ADA (n = 38) at weeks 4, 24, and 48 were further evaluated by ANCOVA (∗*P* < .05), with baseline concentration as a covariate. The PBO group (n = 40) crossed over to GUS at week 16. Healthy control sera (n = 25) were included as reference. Error bars represent SEs. ADA, adalimumab; ANCOVA, analysis of covariance; GUS, guselkumab; LS, lesional skin; MDC, macrophage-derived chemokine; PBO, placebo; PD, pharmacodynamic; SE, standard error.

Rapid and sustained reductions from baseline in serum IL-17A, IL-17F, and IL-22 levels were observed with guselkumab at weeks 4, 24, and 48 (all *P* < .001) (Figure 2a–c). Although ADA also significantly reduced serum IL-17A, IL-17F, and IL-22 levels from baseline at weeks 4, 24, and 48 (all *P* < .01), reductions were significantly greater with guselkumab for IL-17A at weeks 4 and 48 (*P* < .05) (Figure 2a); IL-17F at weeks 4, 24, and 48 (all *P* < .05) (Figure 2b); and IL-22 levels at weeks 4 and 48 (*P* < .05) (Figure 2c). Serum IL-23 levels were slightly reduced by ADA at weeks 4 and 48 (all *P* < .01); however, no significant changes were observed with guselkumab (Figure 2d). VOYAGE 2 data have shown that guselkumab treatment has a minimal effect on serum levels of IL-23 between patients who maintained PASI90 response and those who lost PASI 75 response; however, this change was not statistically significant (Gordon et al, 2019).

Both guselkumab and ADA significantly reduced serum CCL22/MDC levels versus baseline at weeks 4, 24, and 48 (all *P* < .001) (Figure 2e); however, there were no significant differences between them. ADA but not guselkumab significantly reduced CCL4/MIP-1β serum levels at weeks 4, 24, and 48 (all *P* < .001) (Figure 2f), which is consistent with targeting TNF-α (Roach et al, 2002). Both treatment groups demonstrated nonsignificant trends for reduction in serum CXCL8/IL-8 from baseline through week 48 (data not shown).

#### Psoriatic skin transcriptomic profiling at baseline

**Figure 3:**
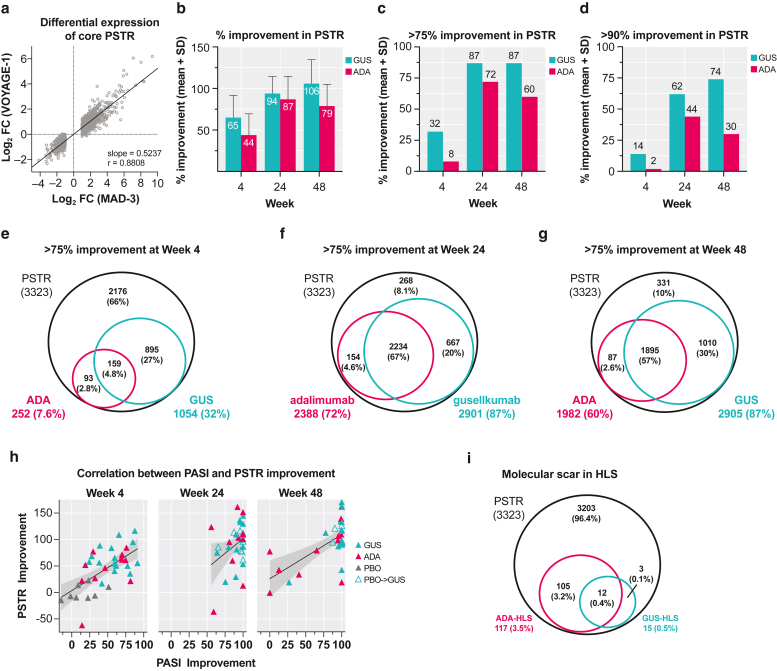
**Psoriatic skin transcriptomic profiling.** (**a**) Association of differential expression of core PSTR genes in this study (VOYAGE 1) versus a previous meta-analysis (MAD-3) (Tian et al, 2012). (**b–d**) Percentage improvement in PSTR and percentage of PSTR with >75% or >90% improvement over time. Venn diagrams of PSTR with >75% improvement at weeks (**e**) 4, (**f**) 24, and (**g**) 48 are shown. (**h**) Correlations between PASI improvement and improvement in PSTR using Pearson’s correlation coefficient (r = 0.68). (**i**) Venn diagram of PSTR at baseline and molecular scars in HLS after treatment with GUS (n = 17) and ADA (n = 13). ADA, adalimumab; FC, fold change; GUS, guselkumab; HLS, healed lesional skin; MAD-3, meta-analysis derived-3; PBO, placebo; PSTR, psoriatic skin transcriptomic signature; SD, standard deviation.

A 1832-gene, core psoriatic skin transcriptomic signature (PSTR) previously identified through a meta-analysis (meta-analysis derived-3 transcriptome) (Tian et al, 2012) was used to assess differential gene expression between LS and NL biopsies at baseline in VOYAGE 1 through microarray profiling. Among these, 1824 (99.6%) genes exhibited the same direction of differential expression in VOYAGE 1 skin samples as shown in the meta-analysis, highlighting the consistency in the PSTR across studies (r = 0.881) (Figure 3a); however, the overall magnitude of differential gene expression was compressed in this study (slope = 0.524) (Figure 3a). This may be due to the use of an Affymetrix chip that utilized only perfect match probes (Affymetrix GeneChip HC HG-U133 Plus perfect match 96-array), compared with previous studies where data were generated using chips with both perfect match and mismatch probes. To accommodate this difference, we chose a fold change (FC) >1.5 (instead of 2) and associated false discovery rate (FDR) <0.05 as selection criteria to distinguish differentially expressed genes (DEG). A total of 3323 individual probe sets, representing 2383 genes, were identified as the PSTR between LS and NL at baseline by paired *t*-test among 37 patients.

#### PD effect on PSTR in response to treatments

Percentage of improvement or normalization in expression of individual probe sets (genes) was used to quantify the PD treatment effect on the PSTR at given time points after treatment, as previously described (Suárez-Fariñas et al, 2011). Overall, percentage improvement in the PSTR progressively increased over time with inhibition of IL-23p19 with guselkumab treatment, whereas improvement in the PSTR peaked at week 24 with ADA. Higher percentage improvements were observed with guselkumab than with ADA at all 3 time points, with larger differences at weeks 4 (65 vs 44%) and 48 (106 vs 79%), suggesting continued improvement over time with IL-23p19 blockade (Figure 3b). Similarly, a larger number of PSTR genes achieved >75 and >90% improvement in expression with guselkumab than with ADA at all 3 time points, with larger differences at weeks 4 (32 vs 8% for >75% improvement and 14 vs 2% for >90% improvement) and 48 (87 vs 60% for >75% improvement and 74 vs 30% for >90% improvement) (Figure 3c and d). The majority of PSTR genes that achieved >75% improvement with ADA (63, 94, and 96% at weeks 4, 24, 48, respectively) also achieved >75% improvement with guselkumab. In contrast, 27, 20, and 30% of PSTR genes that achieved >75% improvement were unique to guselkumab at weeks 4, 24, and 48, respectively, whereas only 3, 5, and 3% of PSTR genes that achieved >75% improvement were unique to ADA at weeks 4, 24, and 48, respectively (Figure 3e–g). Improvement in the PSTR in LS biopsies (n = 102) after treatments was positively correlated with corresponding improvement in PASI (r = 0.68, *P* < .001) (Figure 3h).

The residual disease expression profile or molecular scar for a given treatment was defined as a PSTR gene subset with <75% improvement and remaining FC >1.5 in healed LS (HLS) collected from patients when they achieved a PASI 100 response. As shown in Figure 3i, 3.5% of PSTR genes continued to be differentially expressed in ADA-treated HLS (n = 6) versus only 0.5% in guselkumab-treated HLS (n = 25), with 0.4% of PSTR genes common to both (3.2 vs 0.1% were unique to ADA and guselkumab, respectively). The differences in transcriptional changes reported in Figure 3i might be more directly related to the differences in the treatment mechanisms of action between guselkumab and ADA.

#### Improvement in disease-associated gene expression in PSTR-enriched pathways

**Figure 4:**
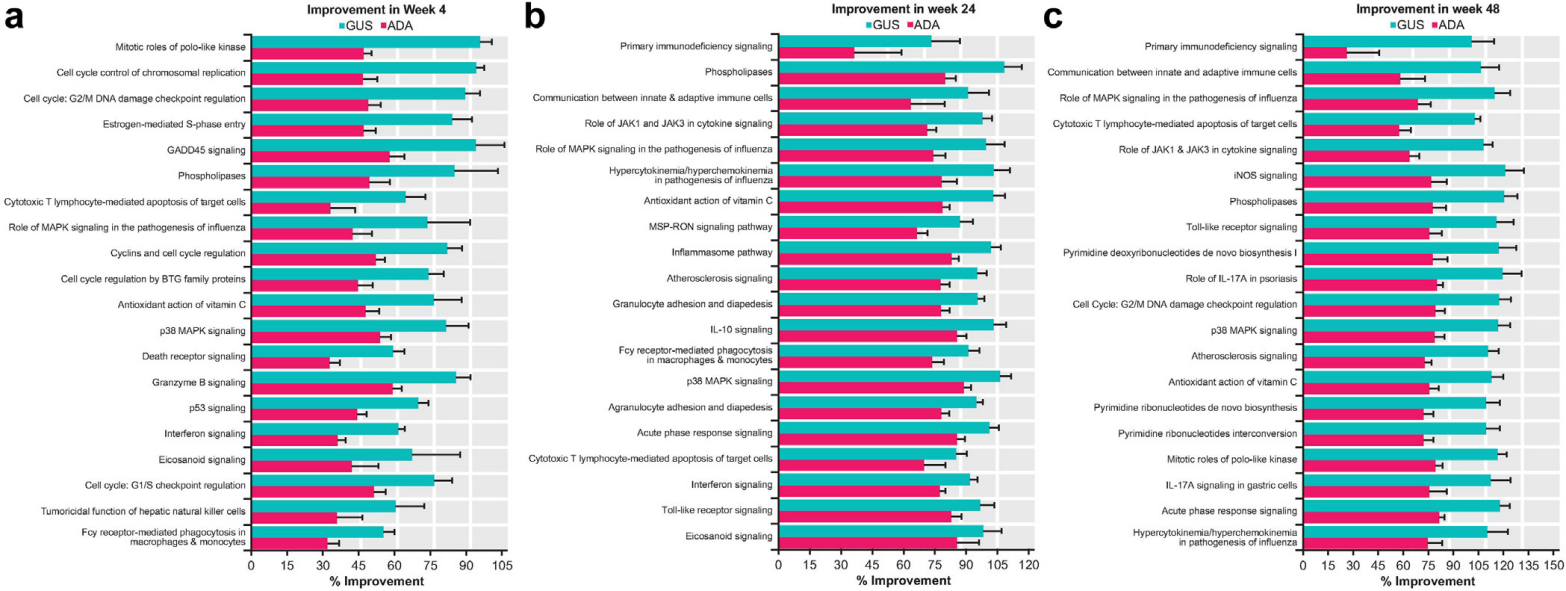
**Normalization of disease-associated gene expression by guselkumab versus adalimumab in PSTR-enriched pathways.** Fifty-six canonical pathways in QIAGEN IPA were selected as enriched with PSTR genes. Percentage improvement (mean + SE) in the PSTR among the top 20 pathways ranked by the difference in improvement between GUS and ADA at weeks (**a**) 4, (**b**) 24, and (**c**) 48. ADA, adalimumab; BTG, B-cell translocation gene; GADD, growth arrest and DNA damage; GUS, guselkumab; iNOS, inducible nitric oxide synthase; IPA, Ingenuity Pathway Analysis; MSP, macrophage-stimulating protein; PSTR, psoriatic skin transcriptomic signature; RON, recepteur d'origine Nantais; SE, standard error.

A total of 56 canonical pathways in the QIAGEN Ingenuity Pathway Analysis software package (QIAGEN, https://digitalinsights.qiagen.com/IPA) were enriched on the basis of the 2383 psoriasis DEGs (FC > 1.5, FDR < 0.05) selected with the following criteria: enriched, with *P* < .01 from hypergeometric enrichment testing; >20% of genes in the psoriasis DEG profile; and >5 genes in the psoriasis DEGs overlapping with the Ingenuity Pathway Analysis pathway (Krämer et al, 2014). Better improvements in the PSTR were observed in the majority of these pathways with guselkumab versus ADA, at all 3 time points assessed (Figure 4 and Table 3).

**Table 3:** Normalization of Disease-Associated Gene Expression by GUS Versus ADA in Psoriasis-Related Pathways^1^

Ingenuity Canonical Pathway, % Improvement	wk 4			wk 24			wk 48		
	ADA	GUS	Difference	ADA	GUS	Difference	ADA	GUS	Difference
Mitotic roles of polo-like kinase	47.2	96.1	48.9	98.6	102.7	4.1	79.5	116.7	37.2
Cell cycle control of chromosomal replication	47.0	94.5	47.5	102.0	103.4	1.3	90.7	123.5	32.8
Cell cycle: G2/M DNA damage checkpoint regulation	49.2	89.7	40.5	90.9	100.9	10.0	79.5	117.7	38.3
Estrogen-mediated S-phase entry	47.3	84.3	37.0	93.6	93.1	-0.5	71.3	101.1	29.8
GADD45 signaling	58.1	94.2	36.1	97.2	108.6	11.4	82.5	114.2	31.7
Phospholipases	49.6	85.4	35.8	80.1	108.5	28.4	77.8	120.6	42.7
Cytotoxic T lymphocyte–mediated apoptosis of target cells	33.3	64.8	31.5	70.0	85.3	15.2	57.7	102.9	45.3
Role of MAPK signaling in the pathogenesis of influenza	42.6	74.0	31.4	74.3	99.6	25.3	68.9	115.0	46.1
Cyclins and cell cycle regulation	52.3	82.3	30.0	91.5	96.3	4.8	76.6	107.6	31.0
Cell cycle regulation by BTG family proteins	44.8	74.5	29.6	97.3	99.5	2.2	76.8	107.3	30.5
Antioxidant action of vitamin C	48.1	76.7	28.6	78.7	103.1	24.4	75.8	113.1	37.4
p38 MAPK signaling	54.2	81.7	27.6	89.1	106.3	17.2	78.9	117.0	38.1
Death receptor signaling	32.9	59.6	26.7	81.1	91.9	10.7	72.0	91.5	19.5
Granzyme B signaling	59.4	85.9	26.4	96.4	100.1	3.7	80.1	111.7	31.6
p53 signaling	44.5	70.1	25.6	88.2	93.5	5.3	82.2	108.8	26.6
Interferon signaling	36.3	61.8	25.5	77.5	92.0	14.4	79.0	96.6	17.7
Eicosanoid signaling	42.3	67.6	25.3	85.8	98.3	12.5	75.9	109.2	33.3
Cell cycle: G1/S checkpoint regulation	51.6	76.8	25.3	87.5	90.3	2.7	75.4	98.6	23.2
Tumoricidal function of hepatic natural killer cells	36.0	60.7	24.7	81.8	91.0	9.2	61.6	96.9	35.3

Abbreviations: ADA, adalimumab; BTG, B-cell translocation gene; GUS, guselkumab.

1 56 IPA pathways enriched with 2383 psoriasis DEG (FC > 1.5, FDR < 0.05) were selected with the following criteria: enriched *p* < 0.01; >20% of genes in psoriasis DEG profile; >5 genes in psoriasis DEG overlap with the IPA pathway.

The largest early treatment differences (guselkumab minus ADA percentage improvement at week 4) were observed for pathways involved in cell cycle control, hyperplasia, and altered differentiation that characterize psoriasis (ie, mitotic roles of polo-like kinase [49%], chromosomal replication cell cycle [48%], GADD45 signaling [36%], G2/M DNA damage cell cycle [41%], granzyme B signaling [26%], phospholipases [36%], estrogen-mediated S-phase entry [37%], cyclins and cell cycle regulation [30%], p38 MAPK signaling [28%], and G1/S checkpoint cell cycle [25%]) (Figure 4a and Table 3).

Predominant treatment differences in immune cell signaling and activation pathways at week 48 corroborated findings for overall PSTR genes, indicating sustained normalization of disease-associated gene expression with guselkumab, whereas effects associated with ADA waned after week 24 (Figure 4b and Table 3). Specifically, the largest treatment differences (guselkumab minus ADA percentage improvement at week 48) were observed for pathways related to primary immunodeficiency signaling (75%), communication between innate and adaptive immune cells (48%), role of MAPK signaling in influenza pathogenesis (46%), cytotoxic T lymphocyte–mediated apoptosis of target cells (45%), role of Jak1 and Jak3 in cytokine signaling (45%), inducible nitric oxide synthase signaling (44%), phospholipases (43%), toll-like receptor signaling (40%), pyrimidine deoxyribonucleotides de novo synthesis (40%), and role of IL-17A in psoriasis (39%) (Figure 4c and Table 3).

#### Improvement in disease-associated gene expression in immune/skin inflammation gene signatures

**Figure 5:**
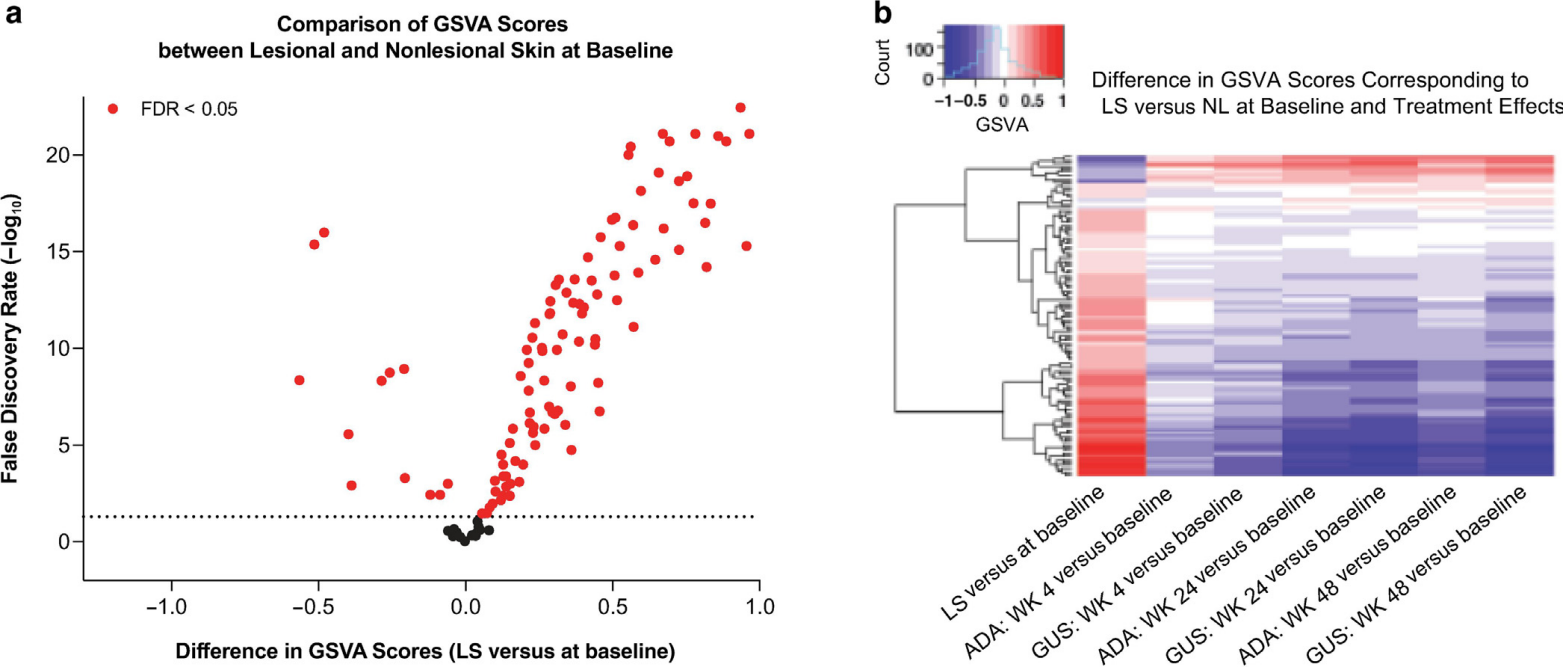
**Treatment effect on immune/skin inflammation gene sets by GSVA.** (**a**) Volcano plot comparing GSVA scores of 124 gene sets between LS and NL at baseline. The 79 gene sets enriched with psoriasis DEG^1^ between LS and NL at baseline are highlighted in red. Dashed horizontal line indicates an FDR = 0.05. Gray dots represent the remaining gene sets not enriched by psoriasis DEG. (**b**) Heatmap of difference in GSVA scores in 108 gene sets (rows), corresponding to comparisons (columns). ^1^Gene set with ≥6% and >20% of genes differentially expressed (FC > 1.5) between LS and NL at baseline. ADA, adalimumab; DEG, differentially expressed gene; FC, fold change; FDR, false discovery rate; GSVA, gene set variation analysis; GUS, guselkumab; LS, lesional skin; NL, nonlesional skin; WK, week.

We further evaluated immune and skin inflammation gene signatures reported to be associated with psoriasis (Brodmerkel et al, 2019). Overall differential expression between LS and NL at baseline was evaluated in 124 curated gene sets by gene set variation analysis (GSVA). A total of 108 gene sets exhibited significant differences (FDR < 0.05) in GSVA scores between LS and NL biopsies (Figure 5a), whereas differences in GSVA scores between LS and NL biopsies at baseline were more efficiently normalized by guselkumab than by ADA at each follow-up time point (Figure 5b).

**Figure 6:**
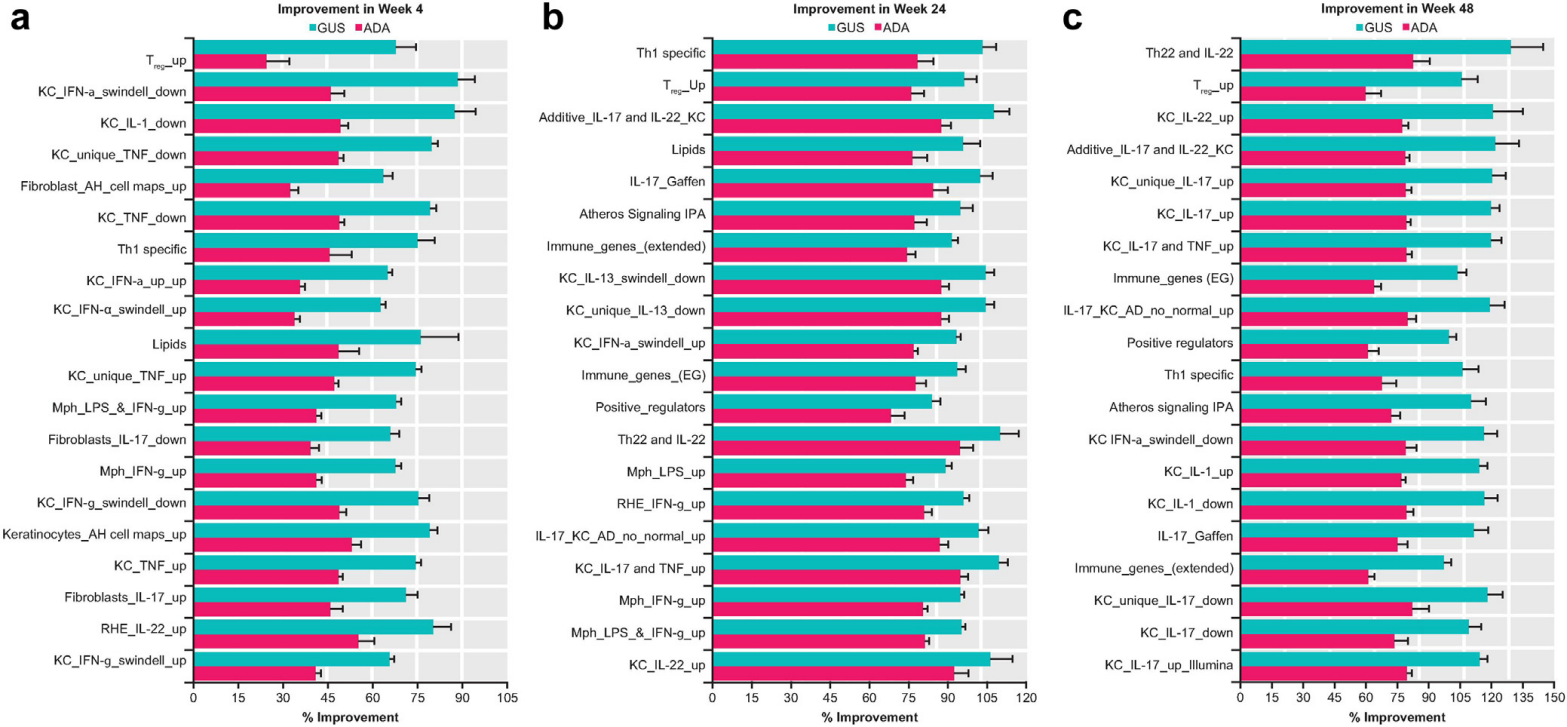
**Normalization of disease-associated gene expression by GUS versus ADA in immune cell and skin inflammation gene sets.** GSVA was used to evaluate differential expression of 124 gene sets between baseline LS and NL. Percentage improvement (mean + SE) in the PSTR among the top 20 sets ranked by the difference in improvement between GUS and ADA at weeks (**a**) 4, (**b**) 24, and (**c**) 48 is shown. AH gene sets are from (Asifa) (Haider et al, 2008). AD, atopic dermatitis; ADA, adalimumab; Atheros, atherosclerosis; GSVA, gene set variation analysis; GUS, guselkumab; IPA, Ingenuity Pathway Analysis; KC, keratinocyte; LPS, lipopolysaccharide; LS, lesional skin; Mph, macrophage; NL, nonlesional skin; PSTR, psoriatic skin transcriptomic signature; RHE, epithelial cell; SE, standard error; Th, T helper; T_reg_, regulatory T cell.

Among these, 71 gene sets were further selected as enriched with PSTR genes (on the basis of FDR < 0.05 from a hypergeometric enrichment test and >20% in psoriasis DEG profile and >5 in the PSTR gene set). Consistent with findings in Ingenuity Pathway Analysis pathways and GSVA results, better improvements in the PSTR were observed with guselkumab than with ADA in almost all of these gene sets, at all 3 time points, with larger differences at weeks 4 and 48 (Figure 6 and Table 4).

**Table 4:** Normalization of Disease-Associated Gene Expression by GUS Versus ADA in Immune Cell and Skin Inflammation Gene Sets (n = 71)^1^

Gene set, % improvement	Cell type	Week 4			Week 24			Week 48		
		ADA	GUS	Difference	ADA	GUS	Difference	ADA	GUS	Difference
T_reg__up_	T_reg_	24.7	68.0	43.3	76.1	96.4	20.4	60.0	105.9	45.9
KC_IFN-α_swindell_down_	Keratinocyte	46.2	88.7	42.5	88.1	101.5	13.4	79.2	116.7	37.5
KC_IL-1_down_	Keratinocyte	49.4	87.6	38.2	87.8	100.9	13.2	79.5	116.8	37.3
KC_unique_TNF_down_	Keratinocyte	48.8	79.9	31.1	92.7	98.2	5.5	82.7	111.6	28.9
Fibroblast_AH cell maps_up_	Fibroblast	32.7	63.8	31.1	95.2	92.4	-2.8	88.0	108.2	20.2
KC_TNF_down_	Keratinocyte	49.0	79.4	30.3	92.3	97.9	5.6	82.2	111.2	29.0
Th1 specific_	Th1	45.8	75.3	29.4	78.5	103.5	25.0	67.8	106.4	38.6
KC_IFN-α_up_up_	Keratinocyte	35.9	65.2	29.3	79.7	92.3	12.6	76.1	79.9	3.8
KC_IFN-α_swindell_up_	Keratinocyte	34.0	62.9	28.9	77.1	93.4	16.4	74.3	87.2	12.9
Lipids_		48.8	76.3	27.5	76.6	96.0	19.4	74.1	108.4	34.3
KC_unique_tnf_up_	Keratinocyte	47.3	74.6	27.3	88.6	97.4	8.7	79.7	110.3	30.6
Mph_LPS_&_IFN-γ_up_	Macrophage	41.3	68.1	26.7	81.4	95.5	14.1	71.9	98.9	27.0
Fibroblasts_IL-17down_	Fibroblast	39.4	66.2	26.7	86.3	93.0	6.7	83.8	91.7	7.9
Mph_IFN-γ up_	Macrophage	41.3	67.9	26.6	80.7	95.0	14.3	72.1	97.6	25.5
KC_IFN-γ_swindell_down_	Keratinocyte	49.1	75.6	26.5	90.8	92.5	1.6	81.8	106.0	24.2
Keratinocytes_AH cell maps_up_	Keratinocyte	53.3	79.3	25.9	93.7	98.8	5.1	83.7	113.0	29.3
KC_TNF_up_	Keratinocyte	48.8	74.6	25.8	89.3	98.6	9.3	79.7	111.3	31.6
Fibroblasts_IL-17_up_	Fibroblast	46.1	71.4	25.3	86.8	94.0	7.1	80.4	98.5	18.0
RHE_IL-22_up_	Epithelial cell	55.5	80.4	25.0	92.6	102.6	10.0	78.0	108.0	30.0
KC_IFN-γ_swindell_up_	Keratinocyte	41.0	65.8	24.8	80.3	93.6	13.3	74.6	98.4	23.9
IFN-γ effect_in_normal_JID2012_up_		41.5	66.1	24.6	80.9	94.4	13.5	70.5	100.9	30.4
Mph_LPS_up_	Macrophage	33.4	57.4	24.1	74.1	89.3	15.2	72.4	83.0	10.7
Fibroblasts_IFN-γ_up_	Fibroblast	42.0	65.6	23.7	85.4	94.8	9.4	79.0	101.7	22.6
KC_IFN-γ_up_	Keratinocyte	46.7	70.3	23.6	88.8	96.2	7.4	80.4	106.2	25.7
KC_TNF_swindell_down_	Keratinocyte	48.4	72.0	23.6	92.1	91.8	-0.2	84.7	105.2	20.6
T_reg__Act_up	T_reg_	38.3	61.7	23.4	80.7	92.6	11.9	71.1	101.9	30.8
KC_IL-22_up_	Keratinocyte	56.1	78.9	22.8	92.6	106.5	13.9	77.4	120.9	43.5
RHE_IFN-γ_up_	Epithelial cell	39.2	61.8	22.6	81.1	96.2	15.1	74.4	105.6	31.2
KC_IL-17_swindell_down_	Keratinocyte	43.4	65.7	22.3	86.5	93.4	6.9	78.9	95.5	16.7
MyoFbs_IL-23A		46.8	68.7	21.9	84.7	98.3	13.6	72.5	106.5	33.9
Lipids_sphingolipids_		48.4	70.0	21.6	79.8	91.3	11.5	78.1	98.3	20.1
T_reg__Act_down	T_reg_	33.9	55.2	21.3	77.7	82.5	4.8	63.8	98.3	34.5
EOS_IL-13_down_	Eosinophils	45.6	66.7	21.1	90.8	99.5	8.7	83.2	106.3	23.1
EOS_IL-13_up_	Eosinophils	45.3	66.3	21.0	86.0	92.4	6.4	74.0	103.9	29.8
KC_IL-4_swindell_down_	Keratinocyte	38.6	59.4	20.9	79.8	91.8	12.0	73.2	90.4	17.3
KC_unique_IL-4_down_	Keratinocyte	38.6	59.4	20.9	79.8	91.8	12.0	73.2	90.4	17.3
Atheros signaling IPA_		41.6	62.2	20.6	77.4	95.0	17.7	72.3	110.6	38.2
MigDC_human_up_vs xpress_NAnanJEM04_	Dendritic cell	46.5	66.6	20.2	85.7	93.2	7.6	74.8	104.1	29.3
IFN-γ effect_in_normal_JID2012_down_		38.7	58.6	20.0	86.1	93.0	7.0	79.9	104.1	24.2
IL-17_KC_AD_no_normal_down_	Keratinocyte	38.9	58.6	19.7	86.0	94.6	8.5	81.6	99.9	18.3
Melanocytes_down_		46.9	66.7	19.7	85.7	88.9	3.3	71.6	98.7	27.1
Immune_genes_(extended)_		37.4	57.0	19.6	74.5	91.6	17.1	61.3	97.6	36.3
RHE_IL-17_down_	Epithelial cell	50.1	69.3	19.2	90.3	96.1	5.8	81.7	105.2	23.5
T_cells_up_	T cell	54.3	73.5	19.2	91.4	94.5	3.1	79.8	106.0	26.2
Positive_Regulators_		31.6	50.7	19.2	68.4	84.0	15.6	61.1	99.9	38.8
KC_TNF_up_Illumina_	Keratinocyte	46.5	65.0	18.6	83.1	96.2	13.1	76.4	104.5	28.0
RHE_IL-17_up_	Epithelial cell	55.7	73.2	17.5	92.9	100.7	7.8	84.2	113.1	28.8
KC_IL-13_swindell_down_	Keratinocyte	43.3	60.7	17.5	87.7	104.7	17.0	82.3	115.3	33.0
KC_unique_IL-13_down_	Keratinocyte	43.3	60.7	17.5	87.7	104.7	17.0	82.3	115.3	33.0
KC_TNF_swindell_up_	Keratinocyte	50.3	67.4	17.1	90.3	98.1	7.8	78.7	110.2	31.5
KC_IL-17_swindell_up_	Keratinocyte	53.7	70.3	16.6	95.0	104.5	9.5	84.1	118.8	34.7
Normal_basalvsepidermis_down_		52.2	68.2	16.0	92.6	97.5	4.9	83.3	110.7	27.4
Synergistic_IL-17 and TNF-α_KC_	Keratinocyte	51.8	67.1	15.3	87.5	98.1	10.6	79.4	109.3	29.9
Immune_genes_(EG)_		44.8	59.5	14.7	77.8	93.8	15.9	64.1	103.9	39.8
IL-17_KC_AD_no_normal_up_	Keratinocyte	52.4	66.6	14.2	87.0	102.0	15.0	80.0	119.6	39.6
Additive_IL-17 and IL-22_Kc_	Keratinocyte	55.3	68.9	13.6	87.7	107.8	20.1	79.1	122.1	43.0
KC_unique_IL-17_up_	Keratinocyte	58.0	71.4	13.4	93.6	103.4	9.8	79.3	120.5	41.2
Additive_IL-17 and TNF-α_KC_	Keratinocyte	55.7	68.9	13.2	91.7	99.2	7.5	82.6	114.6	32.0
KC_IL-17_up_	Keratinocyte	60.4	73.3	12.9	94.5	107.7	13.2	79.5	120.1	40.6
KC_IL-17 and TNF_up_	Keratinocyte	61.6	74.2	12.6	94.9	109.8	14.8	79.6	119.9	40.4
Synergistic_IL-17 and IL-22_KC_	Keratinocyte	56.3	68.6	12.2	90.5	102.1	11.6	81.7	108.9	27.2
KC_IL-1_up_	Keratinocyte	53.8	64.2	10.4	91.7	101.6	9.9	77.1	114.3	37.3
KC_IL-22_down_	Keratinocyte	55.8	66.1	10.3	82.8	94.4	11.6	79.8	108.1	28.3
KC_IL-17_up_Illumina_	Keratinocyte	58.2	68.0	9.9	94.2	104.2	10.0	79.8	114.5	34.7
Th22 and IL-22_	Th22	56.0	65.8	9.8	94.8	110.2	15.4	82.8	129.5	46.7
Th17_	Th17	58.9	68.1	9.2	91.0	101.8	10.7	75.0	108.5	33.5
Th17 specific_	Th17	58.9	68.1	9.2	91.0	101.8	10.7	75.0	108.5	33.5
IL-17_Gaffen_		55.8	63.3	7.6	84.5	102.5	18.0	75.2	111.8	36.6
KC_unique_IL-17_down_	Keratinocyte	72.1	75.5	3.4	93.7	101.5	7.8	82.4	118.3	35.9
KC_IL-17_down_	Keratinocyte	65.8	68.3	2.5	86.4	94.1	7.7	73.7	109.4	35.7
Mph_TNF-α_up_	Macrophage	26.4	20.5	-5.9	67.3	71.5	4.2	69.8	88.3	18.5

Abbreviations: AD, atopic dermatitis; ADA, adalimumab; Atheros, atherosclerosis; EOS, eosinophils; FC, fold change; FDR, false discovery rate; GUS, guselkumab; GSVA, gene set variation analysis; IFN, interferon; IL, interleukin; IPA, Ingenuity Pathway Analysis; JID, Journal of Investigative Dermatology; KC, keratinocyte; LPS, lipopolysaccharide; LS, lesional skin; Mph, macrophage; MigDC, migratory dendritic cell; MyoFb, myofibroblast; NL, nonlesional skin; PSTR, psoriatic skin transcriptomic signature; RHE, epithelial cell; Th, T helper; TNF, tumor necrosis factor; T_reg_, regulatory T cell.

AH gene sets from (Asifa) Haider et al, 2008.

1 The differential expression in 124 curated gene sets related to immune cell and skin inflammation between LS and NL at baseline were evaluated by GSVA. 71 gene sets were selected based on having significant differences in GSVA scores (FDR <0.05) and with ≥6% and >20% of genes in PSTR (2383 genes with FC >1.5).

The largest early treatment differences at week 4 were observed among gene signatures modulated by IFN-α, IL-1, and TNF-α in keratinocytes (Figure 6a and Table 3), whereas the largest treatment differences at week 48 were observed among gene signatures modulated by IL-17 and IL-22 (Figure 6c and Table 3). Better improvement in genes enriched for regulatory T cells were detected with guselkumab than with ADA as early as week 4 and were sustained through week 48 (Pfoertner et al, 2006).

#### qRT-PCR results

**Figure 7:**
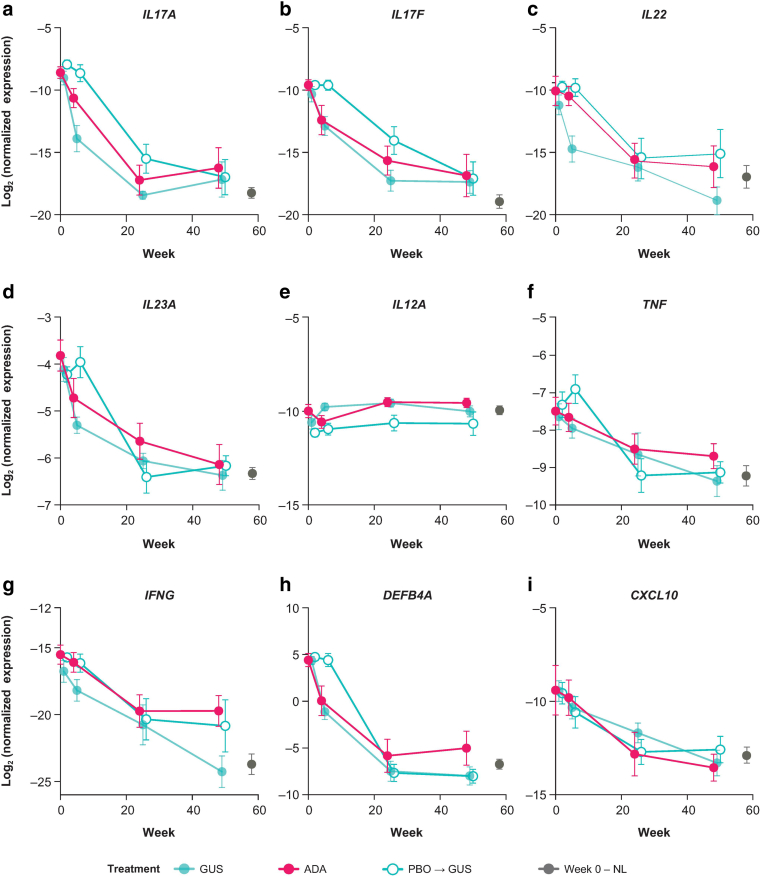
**GUS-treated patients showed faster and greater reductions of IL-17a and IL-22 expression than ADA-treated patients.** Results from 3 of the rerun genes, including (**a**) *IL17A*, (**b**) *IL17F*, and (**c**) *IL22* plus 6 other genes that included (**d**) *IL23A*, (**e**) *IL12A*, (**f**) *TNF*, (**g**) *IFNG*, (**h**) *DEFB4A*, and (**i**) *CXCL10*, profiled using Fluidigm RT-PCR panel (mean + SE). The y-axis (log_2_ normalized expression) was calculated as negative delta ct to the ct values of the reference probe in the qPCR panel. GUS treated, n = 17; ADA treated, n = 13; PBO to GUS, n = 8. ADA, adalimumab; GUS, guselkumab; NL, nonlesional skin; PBO, placebo; SE, standard error.

We used qRT-PCR to confirm and extend observations from the skin gene expression profiles determined by microarray analyses. A total of 121 genes from 38 skin biopsy samples (guselkumab, n = 17; ADA, n = 13; placebo to guselkumab, n = 8) were profiled using a Fluidigm Real-Time PCR panel (complete results are provided in Table 5). The baseline LS versus NL differences and weeks 4, 24, and 48 LS FC versus baseline within the 3 treatment groups are summarized in Table 6. Six genes from the Fluidigm RT-PCR panel failed quality control and were reprofiled using a standard qRT-PCR method. Results from 3 of the rerun genes (*IL17A*, *IL17F*, and *IL22*) plus 6 other genes (*IL23A*, *IL12A*, *TNF*, *IFN*γ, *DEFB4A*, and *CXCL10*) profiled using the Fluidigm RT-PCR panel are summarized in Figure 7a–i. Findings show that guselkumab treatment led to reductions in both IL-17A and IL-22 expression as early as week 4, whereas ADA showed almost no effect on IL-22 expression and minimal (nonsignificant) reductions of IL-17A at the same early time point.

**Table 5:** Full qPCR Results: Baseline LS Versus NL Differences and FC (vs Baseline) by Different Treatment Groups^1^^,^^2^

Gene Name	Category	Entrez ID	Baseline	PBO → GUS			GUS			ADA		
			LS Versus NL	wk4	wk24	wk48	wk4	wk24	wk48	wk4	wk24	wk48
*DEFB4A*	Tissue inflammation	1673 /// 100289462	>1000	1.09	< −1000	< −1000	−52.66	< −1000	< −1000	−29.82	< −1000	−525.79
*S100A7A*	Tissue inflammation	338324	1007.58	1.03	−670.01	−2421.81	−25.70	−5446.86	−7519.61	−12.30	−587.01	−252.82
*S100A8*	Tissue inflammation	6279	695.30	1.40	−772.21	−989.69	−19.46	< −1000	< −1000	−13.23	−577.00	−161.08
*S100P*	Tissue inflammation	6286	8.24	1.55	−19.10	−11.91	−4.18	−7.62	−16.20	−5.42	−13.43	−10.02
*S100A7*	Tissue inflammation	6278	−142.32	< −1000	−22.67	−25.84	3.18	1.10	−1.37	< −1000	< −1000	< −1000
*IFNG*	Th1-regulated cytokines	3458	323.03	−1.13	−39.63	−42.05	−4.79	−37.37	−778.34	−1.50	−12.37	−19.67
*IRF7*	Th1-regulated cytokines	3665	26.17	1.73	−350.67	−100.79	−6.54	−186.09	−69.02	−2.87	−8.12	−11.67
*MX1*	Th1-regulated cytokines	4599	13.34	1.27	−10.65	−12.14	−4.84	−11.88	−12.61	−2.60	−9.31	−9.56
*CCL5*	Th1-regulated cytokines	6352	3.43	1.07	−2.03	−2.92	−1.08	−1.97	−2.66	−1.05	−1.71	−1.39
*IL12RB2*	Th1-regulated cytokines	3595	3.41	1.22	−3.31	−3.75	−1.82	−3.03	−3.86	−1.63	−2.96	−3.28
*PRKCQ*	T-cell activation	5588	>1000	1.86	< −1000	< −1000	−20.56	−289.08	< −1000	−26.38	< −1000	< −1000
*CXCR2*	Neutrophil receptor	3579	10.90	1.16	−17.71	−13.63	−4.18	−8.50	−13.35	−3.66	−7.92	−5.70
*CXCR1*	Neutrophil receptor	3577	2.34	−1.37	−9.50	−5.77	−2.59	1.01	−4.89	−2.16	−2.67	−2.40
*CXCL10*	Immune-related pathways	3627	11.43	−1.38	−8.62	−7.60	−2.14	−4.48	−17.07	−2.14	−8.60	−13.14
*LAMP3*	Immune-related pathways	27074	6.32	1.53	−7.75	−10.07	−2.24	−4.64	−9.10	−1.84	−5.29	−6.64
*IRF9*	Immune-related pathways	10379	3.02	4.95	−2.81	−1.87	−1.49	−3.90	−3.96	−1.15	−1.75	−5.28
*FOXP3*	Immune-related pathways	50943	2.05	1.09	−2.09	−1.44	1.10	−1.10	−3.22	1.18	−1.21	−1.52
*CCR6*	Immune-related pathways	1235	1.97	1.17	−1.77	−1.90	−1.06	−1.35	−2.65	1.15	−1.66	−1.88
*CD274*	Immune regulatory (Negative)	29126	11.11	1.11	−8.18	−9.41	−3.69	−7.49	−10.64	−2.60	−7.32	−6.61
*CTLA4*	Immune regulatory (Negative)	1493	7.32	1.49	−7.73	−11.17	−1.60	−2.36	−6.92	−1.08	−5.90	−5.60
*IKBKE*	Immune regulatory (Negative)	9641	2.85	1.54	−4.09	−2.96	−1.48	−3.27	−4.46	−1.20	−2.49	−3.07
*PDCD11*	Immune regulatory (Negative)	22984	2.11	1.98	−2.10	−1.80	1.07	−1.40	−1.58	−1.58	−1.81	−3.21
*CD69*	Immune regulatory (Negative)	969	1.94	−4.05	−5.47	2.15	1.70	5.42	2.39	2.12	−1.35	−1.48
*IL17A*	IL-23/Th17 axis	3605	755.96	−1.63	−189.60	-528.14	−29.12	−676.24	−282.67	−4.14	−396.56	−151.18
*IL17F*	IL-23/Th17 axis	112744	503.88	−1.00	−21.72	−180.12	−5.85	−123.17	−131.65	−7.00	−66.66	−106.71
*IL19*	IL-23/Th17 axis	29949	>1000	1.72	< −1000	< −1000	−43.50	< −1000	< −1000	−147.79	< −1000	−224.09
*CXCL1*	IL-23/Th17 axis	2919	94.93	−1.00	−146.17	−150.04	−13.58	−45.20	−65.03	−13.13	−84.06	−65.55
*CCL20*	IL-23/Th17 axis	6364	19.24	1.82	−17.35	−37.91	−7.71	−8.42	< −1000	−6.41	−20.66	−104.59
*IL6*	IL-23/Th17 axis	3569	6.48	1.10	−5.70	−3.34	−3.91	−4.61	−5.14	−3.31	−4.20	−4.81
*CCL22*	IL-23/Th17 axis	6367	5.33	1.15	−7.21	−8.70	−2.60	−4.95	−14.20	−1.69	−5.67	−4.36
*IL23A*	IL-23/Th17 axis	51561	4.86	1.32	−4.76	−3.96	−2.33	−3.97	−4.71	−2.02	−4.04	−5.26
*CSF2*	IL-23/Th17 axis	1437	4.47	1.20	−6.78	−15.39	−2.59	−7.33	−5.44	−2.55	−4.24	−5.09
*TNF*	IL-23/Th17 axis	7124	3.26	1.47	−3.97	−3.20	−1.28	−2.13	−3.31	−1.31	−2.09	−2.33
*IL33*	IL-23/Th17 axis	90865	2.28	1.25	−2.41	−2.47	−1.49	−1.67	−2.07	−1.06	−1.84	−1.84
*IL17RC*	IL-23/Th17 axis	84818	1.90	1.28	−2.35	−2.08	−1.79	−2.22	−2.54	−1.34	−2.16	−2.08
*IL17RA*	IL-23/Th17 axis	23765	1.50	1.31	−1.73	−1.73	−1.02	−1.16	−1.70	−1.20	−1.44	−1.66
*RORC*	IL-23/Th17 axis	6097	-5.41	1.10	2.08	2.88	1.75	9.39	2.07	1.95	2.34	2.00
*IFI27*	IFN-α pathway	3429	24.16	1.19	−18.14	−29.30	−4.18	−16.87	−20.81	−3.01	−10.16	−12.29
*IFI44L*	IFN-α pathway	10964	19.47	1.45	−7.96	−6.81	−2.11	−5.88	−5.12	−1.85	−6.22	−15.88
*IFI44*	IFN-α pathway	10561	5.48	1.33	−3.98	−4.84	−1.69	−3.27	−4.32	−1.47	−3.54	−4.58
*MX2*	IFN-α pathway	4600	4.89	1.33	−4.66	−4.51	−2.61	−3.09	−3.54	−1.73	−4.34	−4.74
*IFIT1*	IFN-α pathway	3434	4.70	1.45	−6.21	−4.78	−1.98	−3.61	−3.55	−1.84	−4.42	−6.16
*IFI27L2*	IFN-α pathway	83982	2.96	1.24	−4.22	−3.79	−1.87	−2.82	−4.11	−1.30	−1.62	−2.08
*IL1RL2*	Epithelial IL-36 receptor	8808	1.77	1.49	−2.55	−1.70	−1.11	−1.89	−2.93	−1.13	−1.69	−2.19
*LCE3B*	Epidermal hyperplasia	353143	>1000	1.39	< −1000	< −1000	−113.17	< −1000	< −1000	−91.58	−200.05	−366.28
*LCE3C*	Epidermal hyperplasia	353144	>1000	1.22	< −1000	< −1000	< −1000	< −1000	< −1000	< −1000	< −1000	−497.36
*IL36A*	Epidermal hyperplasia	27179	>1000	1.21	< −1000	< −1000	−192.23	< −1000	< −1000	−161.81	< −1000	< −1000
*DEFB4A*	Epidermal hyperplasia	1673 /// 100289462	>1000	1.13	−5407.83	−6072.71	−52.08	< −1000	< −1000	−29.38	< −1000	−465.68
*LCE3A*	Epidermal hyperplasia	353142	929.18	1.55	< −1000	−962.87	−30.82	−625.01	< −1000	−14.64	−642.60	−172.14
*KRT16*	Epidermal hyperplasia	3868	233.52	−1.42	−434.91	−260.85	−38.45	−113.11	−373.61	−20.66	−88.52	−55.83
*CCL20*	Epidermal hyperplasia	6364	172.44	2.07	−21.23	< −1000	−7.28	−120.82	−282.92	−14.01	< −1000	−325.28
*LCE3E*	Epidermal hyperplasia	353145	49.44	1.33	−41.73	−59.84	−7.20	−59.94	−121.05	−5.55	−22.48	−19.33
*LCE3D*	Epidermal hyperplasia	84648	41.12	1.33	−46.45	−79.57	−7.32	−70.27	−163.41	−4.13	−21.39	−22.60
*KRTAP4-4*	Epidermal hyperplasia	84616	7.62	1.48	−17.20	1.93	2.53	−1.69	−1.31	−7.54	−20.41	−1.35
*HBEGF*	Epidermal hyperplasia	1839	7.56	−1.11	−8.10	−7.06	−4.51	−6.93	−8.01	−2.67	-5.26	−4.53
*AREG*	Epidermal hyperplasia	374	5.71	1.04	−15.43	−5.45	−3.60	−4.95	−6.27	−3.31	-5.42	−5.24
*TGFA*	Epidermal hyperplasia	7039	5.42	1.32	−6.69	−6.13	−3.63	−5.58	−8.16	−2.40	-4.99	−4.92
*IL36B*	Epidermal hyperplasia	27177	4.04	−1.17	−6.33	−5.36	−3.18	−1.34	−4.18	−2.72	-4.67	−3.15
*45_CALM1*^3^	Epidermal hyperplasia	801	2.96	1.02	−3.34	−3.69	−2.28	−3.43	−3.34	−1.95	-2.93	−2.90
*IL22RA2*	Epidermal hyperplasia	116379	2.81	1.64	−6.16	−5.86	−1.84	−2.94	−30.70	−1.85	-8.90	−3.84
*ADAM10*	Epidermal hyperplasia	102	2.21	1.12	−3.12	−2.41	−1.36	−1.75	−2.38	−1.50	−2.22	−2.64
*KRT33A*	Epidermal hyperplasia	3883	2.13	1.30	−1.82	1.40	−1.52	−1.45	−2.14	−2.31	−2.29	−1.26
*75_CALM1*^3^	Epidermal hyperplasia	801	1.90	1.39	−3.07	−2.03	−1.27	−1.93	−2.19	−1.28	−1.67	−2.25
*IL22RA1*	Epidermal hyperplasia	58985	1.28	2.02	−1.98	−1.18	−1.28	−1.27	−2.48	−1.26	−1.53	−2.53
*KRT18*	Epidermal hyperplasia	3875	−1.72	1.70	1.72	2.25	2.07	1.41	1.58	1.38	1.96	1.27
*KRT73*	Epidermal hyperplasia	319101	−3.55	1.58	2.53	5.71	4.63	5.86	2.30	4.37	4.88	1.57
*APOC1*	Comorbidities: Cardiovascular	341	−2.12	1.33	−1.13	1.81	1.76	1.20	−1.06	−1.21	1.44	1.33
*GAPDH*	Assay Control	2597	2.08	1.30	−3.65	−2.38	−1.43	−2.02	−2.73	−1.36	−1.51	−2.09
*18S*	Assay Control		1.79	1.18	−6.20	−3.35	−1.54	−2.29	−2.08	1.14	−1.44	−1.38
*RPLP0*	Assay Control	6175	1.76	1.39	−2.83	−2.07	−1.22	−1.37	−2.22	−1.37	−1.58	−2.14
*IL21*		59067	>1000	1.47	< −1000	< −1000	−534.85	< −1000	< −1000	−13.82	< −1000	< −1000
*TF*		7018	−1.36	1.41	−1.17	1.10	1.32	1.21	1.31	−1.18	−1.13	1.13
*HLA-DQA1*		3117	−1.85	−2.44	2.92	3.97	1.13	5.63	−1.38	−4.03	−1.72	-5.99
*IL20*		50604	574.86	2.37	< −1000	−173.07	−53.24	−365.40	< −1000	−290.02	−124.57	−182.03
*S100A12*	Tissue inflammation	6283	566.75	1.96	−858.97	−782.24	−26.92	< −1000	−844.56	−15.99	−405.01	−218.71
*IL27*		246778	196.26	6.23	−98.57	−258.38	−2.51	−24.03	−4.98	−58.96	−888.19	4.20
*IFNA1*		3439	191.16	−2.85	−1.23	−31.14	13.91	−20.43	−9.79	−1.25	−353.67	−5.58
*IL17C*		27189	64.91	1.44	−114.25	−28.21	−8.87	−12.19	−46.63	−15.24	−762.64	−20.17
*IL23R*	IL-23/Th17 axis	149233	1.78	−1.05	1.16	1.08	−1.52	−2.02	−1.47	1.24	1.07	−1.29
*IL36G*		56300	28.69	1.48	−52.06	−42.29	−4.50	−28.80	−58.22	−4.57	−21.12	−28.10
*ISG15*		9636	21.70	1.29	−16.46	−17.20	−7.83	−15.71	−13.89	−3.69	−14.53	−14.17
*IL1B*		3553	20.66	−1.39	−54.87	−52.15	−8.37	−19.88	−23.71	−12.13	−25.73	−12.41
*IL36RN*		26525	11.69	1.28	−11.86	−13.72	−4.69	−10.88	−21.56	−2.67	−8.96	−10.13
*IL12RB1*		3594	11.39	−1.00	−2.81	−7.76	−2.24	−1.93	−14.58	−1.50	−3.26	−3.31
*CARHSP1*		23589	10.77	1.24	−12.54	−10.92	−5.79	−9.61	−11.36	−3.10	−7.19	−7.30
*CCL4*		6351	10.73	1.17	−11.86	−7.07	−2.49	−5.39	−9.09	−3.16	−6.66	−6.36
*CAMP*		820	10.62	1.17	−71.05	−18.05	−2.23	−2.48	−6.11	−5.72	−10.58	−8.54
*TBX21*		30009	6.14	6.18	−1.05	−2.53	−1.25	−2.68	−8.40	1.13	−1.63	1.25
*SPRR2G*		6706	5.31	1.50	−10.44	−21.11	−1.20	−14.29	−36.63	−1.77	−6.32	−8.56
*TFRC*		7037	4.99	1.05	−15.99	−4.25	−1.94	−3.88	−5.01	−1.31	−1.45	−2.04
*KLK8*		11202	4.91	1.30	−7.03	−7.73	−2.32	−5.38	−10.31	−1.80	−3.84	−5.12
*IL10*		3586	4.53	−1.05	−5.87	−4.92	−1.83	1.14	−4.46	−1.65	−2.65	−2.93
*CARD14*		79092	4.31	1.42	−4.98	−6.19	−2.24	−4.02	−21.37	−1.58	−4.09	−4.33
*TRIM22*		10346	4.20	1.48	−3.40	−3.41	−1.78	−2.73	−4.00	−1.41	−2.65	−3.37
*EBI3*		10148	2.18	−1.30	−1.78	−1.72	−2.43	−1.97	−1.54	−1.47	−2.12	−1.36
*KLRB1*		3820	3.97	−1.14	−2.25	−2.03	1.04	−1.47	−2.03	1.19	−2.01	−2.45
*HMMR*		3161	3.89	1.11	−8.10	−6.46	−3.07	−3.38	−5.69	−1.69	−3.51	−4.36
*WFDC12*		128488	3.61	1.54	−2.90	−4.61	1.06	−4.29	−8.82	1.09	−2.39	−2.90
*CALML5*		51806	3.52	1.35	−5.22	−4.91	−1.75	−5.02	−7.96	−1.35	−2.78	−4.11
*PLA2G2A*		5320	3.21	1.15	−9.66	−5.23	−4.24	−3.21	−4.56	−2.28	−2.27	−1.55
*GPX3*		2878	3.18	1.26	−4.18	−3.18	−3.97	−2.81	−2.79	−2.24	−3.39	−2.90
*HMGB3*		3149	2.95	1.13	−4.50	−4.11	−2.09	−3.02	−3.94	−1.56	−2.77	−3.28
*PMCH*		5367	2.80	1.68	−4.78	−2.93	−1.42	−2.06	−3.29	−1.51	−2.24	−3.09
*CEBPD*		1052	2.45	1.15	−2.92	−2.69	−1.95	−1.94	−2.82	−1.35	−2.30	−2.47
*CD4*		920	2.38	−1.06	−2.00	−2.53	−1.64	−2.19	−2.39	1.01	−1.42	−1.31
*CD8A*		925	2.24	1.15	−2.14	−2.96	−1.48	1.35	−3.56	−1.15	−1.96	−1.87
*ITGAE*		3682	2.14	1.16	−2.59	−2.43	−1.52	−1.71	−2.47	−1.14	−1.80	−1.92
*SELPLG*		6404	1.96	1.36	−2.74	−1.97	−1.14	−1.87	−2.76	1.22	−1.25	−1.78
*IL27RA*		9466	1.67	1.09	−1.86	−1.87	−1.31	−1.08	−2.16	1.11	−1.51	−1.50
*ME1*		4199	1.42	−1.27	−4.26	−1.52	1.49	1.25	−1.53	−2.10	−3.38	−5.17
*C1QTNF3*		114899	1.12	1.75	1.12	1.25	2.00	1.04	−1.08	1.28	1.44	1.25
*ELANE*		1991	−1.02	1.54	−1.39	1.14	1.01	−1.21	−1.54	1.19	1.62	1.04
*PCOLCE2*		26577	−1.08	1.05	1.08	1.29	−1.06	−1.11	−1.01	−1.17	1.47	1.15
*CD1A*		909	−1.20	1.37	1.35	1.14	1.28	1.07	−1.44	2.32	1.14	−1.19
*HBB*		3043	−1.34	1.06	−1.35	1.37	−1.58	−1.06	1.81	−1.70	−2.39	−1.40
*CDON*		50937	−1.35	1.47	−1.14	1.27	1.65	1.20	1.15	1.22	1.28	−1.02
*GATA3*		2625	−1.45	1.20	1.15	1.20	−1.12	−1.04	−1.32	1.68	1.07	1.02
*HBA1 /// HBA2*		3039 /// 3040	−1.48	1.16	−1.60	1.48	−1.41	−1.03	1.94	−1.46	−1.88	−1.24
*IL17RE*		132014	−1.58	1.38	−1.10	1.18	1.19	1.45	−1.34	1.52	1.17	−1.21
*IL12A*		3592	−2.07	1.09	1.28	1.53	1.45	4.04	1.25	−1.30	1.38	1.28
*CD207*		50489	−2.29	1.82	1.95	1.99	3.37	2.09	1.12	3.01	1.43	1.04
*FGF9*		2254	−3.11	1.67	2.02	2.35	1.66	5.57	1.83	1.90	3.32	2.06
*IL22*		50616	84.11	−1.01	−56.18	−42.19	−11.62	−32.92	−215.09	−1.32	−49.06	−57.21

Abbreviations: ADA, adalimumab; AREG, amphiregulin; CAMP, cathelicidin antimicrobial peptide; FC, fold change; GUS, guselkumab; HMMR, hyaluronan mediated motility receptor; ID, identification; LCE, late cornified envelope; LS, lesional skin; NL, nonlesional skin; PBO, placebo; Th, T helper; TF, transferrin.

1 qPCR: baseline LS versus NL differences and FCs by different treatment groups from all 121 qPCR genes assessed using Fluidigm RT-PCR panel are shown.

2 Large FCs are capped at >1000 or < −1000 fold, similar to previous reports (reference: Sofen et al [2014])

3 45_CALM1 and 75_CALM1 used two different qPCR assays for the same gene.

**Table 6:** qPCR: Baseline LS Versus NL Skin Differences and FC (vs Baseline) by Different Treatment Groups^1^^,^^2^^,^^3^

Gene Name	Category	Entrez ID	Baseline	PBO → GUS			GUS			ADA		
			LS Versus NL (n = 38)	wk4 (n = 9)	wk24 (n = 9)	wk48 (n = 9)	wk4 (n = 16)	wk24 (n = 15)	wk48 (n = 14)	wk4 (n = 12)	wk24 (n = 10)	wk48 (n = 10)
*IL17A*	IL-23/T17 axis	3605	**755.96**	−1.63	**−189.60**	**−528.14**	**−29.12**	**−676.24**	**−282.67**	−4.14	**−396.55**	**−151.18**
*IL17F*	IL-23/T17 axis	112744	**503.88**	−1.00	**−21.72**	**−180.12**	*−5.85*	**−123.17**	**−131.65**	−7.00	**−66.66**	**−106.71**
*IL23R*	IL-23/T17 axis	149233	**1.78**	−1.05	1.16	1.08	−1.52	*−2.02*	*−1.47*	1.24	1.07	−1.29
*IL22*	IL-23/T17 axis	50616	**84.11**	−1.01	**−56.18**	**−42.19**	*−11.62*	**−32.92**	**−215.09**	−1.32	**−49.06**	**−57.21**
*IL23A*	IL-23/T17 axis	51561	4.86	1.32	**−4.76**	**−3.96**	**−2.33**	**−3.97**	**−4.71**	*−2.02*	**−4.036**	**−5.26**
*IL12A*	Th1-regulated cytokines	3592	−2.07	1.09	1.28	1.53	1.45	4.04	1.25	−1.30	1.38	1.28
*TNF*	IL-23/T17 axis	7124	3.26	1.47	−3.97	*−3.20*	−1.29	*−2.13*	*−3.31*	−1.31	−2.09	−2.33
*IFNG*	Th1-regulated cytokines	3458	**323.03**	−1.13	−39.63	−42.05	*−4.79*	*−37.37*	**−778.34**	−1.50	−12.37	−19.67
*DEFB4A*	Tissue inflammation	1673	**>1000**	1.09	**< −1000**	**< −1000**	**−52.66**	**< −1000**	**< −1000**	*−29.82*	**< −1000**	**−525.79**
*CXCL10*	Immune-related pathways	3627	**11.43**	−1.38	*−8.62*	*−7.60*	−2.14	**−4.48**	**−17.07**	−2.14	*−8.60*	*−13.14*
*S100A7A*	Tissue inflammation	338324	**>1000**	1.03	**−670.00**	**< −1000**	**−25.70**	**< −1000**	**< −1000**	−12.30	**−587.01**	**−252.82**
*EBI3*		10148	**2.18**	−1.30	−1.78	−1.72	**−2.43**	*−1.97*	*−1.54*	−1.47	*−2.12*	−1.36

Abbreviations: ADA, adalimumab; CXCL, C-X-C motif chemokine ligand; DEFB4A, defensin beta 4A; Entrez ID, Entrez Gene identifier; EBI3, Epstein-Barr virus-induced 3; FC, fold change; FDR, false discovery rate; GUS, guselkumab; ID, identification; LS, lesional skin; NL, nonlesional skin; PBO, placebo; T17, IL-17 producing T; Th1, T helper 1.

1 qPCR: baseline LS versus NL differences and FC by different treatment groups from 6 qPCR reprofiled genes (*IL17A*, *IL17F*, *IL22*, *IL23R*, *S100A7A*, and *EBI3*) plus 6 other genes (*IL23A*, *IL12p35*, *TNFα*, *IFNγ*, *DEFB4*, and *CXCL10*) profiled using the Fluidigm RT-PCR panel are shown. Log_2_ expression profiles of 9 of the listed genes are plotted in Figure 5. FCs from the full list of 121 genes profiled using the Fluidigm RT-PCR panel are provided in Table 5.

2 Large FCs are capped at >1000 or < −1000 fold, similar to previous reports (Sofen et al, 2014).

3 Bold FCs are statistically significant at FDR < 0.01; gray-highlighted FCs have an FDR < 0.05 but >0.01.

## Discussion

We characterized blood and tissue PD responses in individuals with psoriasis treated with IL-23p19 blockade using guselkumab versus TNF-α blockade using ADA over the course of 48 weeks. Serum biomarkers associated with psoriasis and the IL-23/IL-17 and IL-22 immunologic pathways were elevated at baseline and were reduced with guselkumab to a greater extent than with ADA. Aberrant psoriasis-associated gene expression was documented in LS versus NL at baseline, with gene expression normalization occurring as early as week 4 (and progressively sustained over 48 weeks) with guselkumab; the breadth and depth of gene expression normalization were more extensive with guselkumab than with ADA at all time points assessed. These data provide a comprehensive characterization of PD anti-inflammatory responses in human blood and tissue with IL-23p19 and TNF-α inhibition over time using FDA-approved doses of guselkumab and ADA, respectively.

Baseline serum IL-17A, IL-17F, and IL-22 levels were shown to be higher in patients with moderate-to-severe psoriasis than in healthy controls. Serum CCL22/MDC and CCL4/MIP-1β levels also were elevated at baseline in the psoriasis study population versus healthy controls (Table 2 and Figure 1). These data are consistent with previous observations that markers associated with the IL-23/IL-17 pathway (ie, IL-17A, IL-17F, IL-22, and CCL22/MDC) are elevated in serum from patients with psoriasis (Fotiadou et al, 2015; Suárez-Fariñas et al, 2012). IL-23 levels were consistently low in the serum at baseline, and no significant changes in serum IL-23 levels were observed with guselkumab treatment. The Single Molecule Counting Erenna Assay used to measure serum IL-23 only detects free IL-23 and not guselkumab-bound IL-23 (data on file). Therefore, the lack of effect of guselkumab on serum IL-23 is likely not due to interference of guselkumab in the detection of free IL-23. In addition, IL-23 is a local mediator of inflammation by activating IL-23 receptor–positive cells in the tissue microenvironment (Pawlak et al, 2022; Sherlock and Cua, 2021), and serum IL-23 may not be reflective of local IL-23 levels in LS. The enhanced impact on the transcriptomic profile with guselkumab supports a mechanistic basis for differences beyond simply better clinical response. Furthermore, guselkumab treatment led to significantly greater reductions in IL-17A, IL-17F, and IL-22 serum levels than both placebo and ADA treatments. Guselkumab achieved rapid reduction in serum levels of IL-17A and IL-17F, which were significantly lower than at baseline by week 4 and were maintained at weeks 24 and 48. In addition, guselkumab achieved significantly greater reductions in IL-17A and IL-17F levels than placebo at all time points assessed. Similarly, guselkumab achieved significantly greater reduction of IL-17F serum levels at weeks 4, 24, and 48 than ADA, however, at only week 48 for IL-17A. Guselkumab also reduced serum IL-22 levels to a greater degree than ADA at week 48.

In addition to expected reductions in serum levels of effector cytokines associated with the IL-23/IL-17 and IL-22 pathways, other markers associated with psoriasis were also reduced to a greater extent with guselkumab than with placebo, including CCL22/MDC and CXCL8/IL-8, albeit to a moderate extent. However, ADA but not guselkumab decreased serum CCL4/MIP-1β levels, a marker associated with psoriasis and downstream of TNF-α (Pedrosa et al, 2011; Roach et al, 2002). This observation is aligned with the predominant role of the IL-23/IL-17 axis in driving psoriasis pathogenesis, which is selectively antagonized by guselkumab over ADA.

Taken together, these observations suggest that by blocking IL-23, a key regulatory cytokine that is required for the expansion of T17, T helper 22, and group 3 innate lymphoid cells, guselkumab reduces the ability of these cells to produce key effector cytokines. IL-23 also augments the production of IL-22 from group 3 innate lymphoid cells (Teng et al, 2015; Ward and Umetsu, 2014). Group 3 innate lymphoid cells lack rearranged antigen-specific receptors, rely on the transcription factor RORγt, and respond to IL-1β and IL-23; a subset of group 3 innate lymphoid cells is associated with production of IL-17A and IL-22 in psoriasis lesions (Villanova et al, 2014). In addition, IL-23 maintains some regulatory capacity for Tc17 cells (Ciric et al, 2009; Teunissen et al, 2014).

Increased expression of genes induced by IL-17A in keratinocytes and TCR genes, including genes previously determined to be a part of the PSTR or present as a psoriasis molecular scar (Tian et al, 2012), was more efficiently normalized by guselkumab than by ADA at all time points assessed (Figure 4). Better improvement in expression of genes enriched for regulatory T cells by guselkumab than by ADA was detected as early as week 4 and sustained through week 48. This finding is consistent with a recent substudy of the phase 3 ECLIPSE trial that showed better maintenance of efficacy over time with guselkumab than with secukinumab, an IL-17A blocker (Reich et al, 2019). Among CD11c^+^HLA-DR^+^ mononuclear phagocytes, CD64^bright^CD163^−^CD14^bright^CD1c^−^CD1a^−^ inflammatory monocyte-like cells were identified as the predominant IL-23–producing cells in LS, and guselkumab but not secukinumab reduced the relative numbers of tissue-resident memory T cells and maintained the levels of anti-inflammatory regulatory T cells, which may contribute to prolonged maintenance of clinical response observed with guselkumab treatment (Mehta et al, 2021).

A limitation of this study is the challenge in separating normalization of the skin transcriptional profile from clinical improvements. Some of the differences in transcriptional changes at later time points (especially at week 48) might reflect differences in PASI responses. However, at week 4, there were no significant differences in PASI improvements between the guselkumab- and ADA-treated subgroups with skin biopsy samples for biomarker analyses (also shown previously in Blauvelt et al [2017] for all patients); therefore, early transcriptional changes may be independent of and precede clinical responses seen at later time points and may better reflect differences derived from blocking their respective therapeutic targets: IL-23p19 and TNFα (Gordon et al, 2019). In addition, IL-23 is a local mediator of inflammation that functions by activating IL-23 receptor–positive cells in the tissue microenvironment (Pawlak et al, 2022; Sherlock and Cua, 2021), and serum IL-23 may not be reflective of local IL-23 levels in LS. The enhanced impact on transcriptomic profile with guselkumab supports a mechanistic basis for differences beyond simply better clinical response.

In summary, IL-23p19 blockade by guselkumab in patients with psoriasis (vs TNF-α blockade by ADA) more dramatically reduced serum concentrations of cytokines IL-17A, IL-17F, and IL-22, which are associated with the IL-23/IL-17 pathway, thereby limiting the downstream activity of these effector inflammatory cytokines. Similarly, normalization of psoriasis-associated inflammatory genes was greater in LS samples from guselkumab-treated patients than in those from ADA-treated patients. Differences in protein and gene expression signatures with treatment should be interpreted in the context of differences in distribution of clinical responses between compared patient populations and, hence, significant differences to which associated signaling pathways have been suppressed. The molecular PD findings reported in this analysis are consistent with the differential clinical responses observed between guselkumab and ADA treatment in the VOYAGE 1 study and correlate with the depth of therapeutic responses seen with guselkumab versus ADA treatment in patients with moderate-to-severe psoriasis.

## Materials and Methods

### Trial design and participants

VOYAGE 1 (NCT02207231) was a phase 3, randomized, double-blind, multicenter, placebo-controlled, and active-comparator–controlled study of guselkumab in patients (male 72.6%, White 81.7%) with moderate-to-severe plaque-type psoriasis, which has been previously described (Blauvelt et al, 2017). Study participants were adults with a diagnosis of plaque-type psoriasis (with or without psoriatic arthritis) for ≥6 months before the first administration of study drug. Moderate-to-severe plaque-type psoriasis was defined by an Investigators Global Assessment ≥3, PASI ≥12, and an involved body surface area ≥10%. Eligible participants must have been candidates for and may have previously received either systemic therapy or phototherapy for psoriasis.

### Intervention

**Figure 8:**
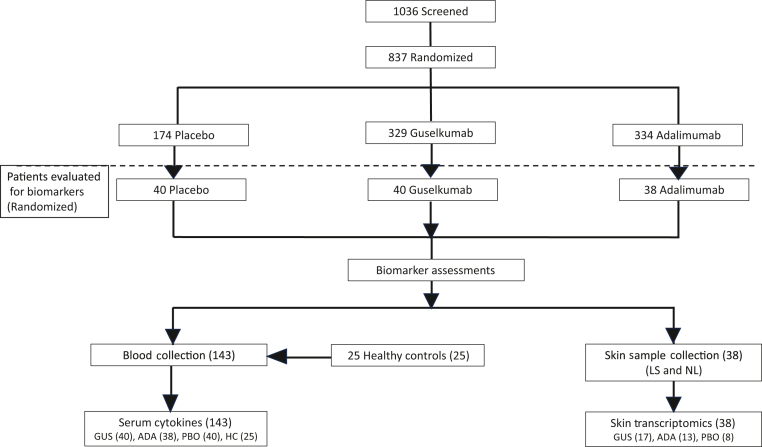
**Consort flowchart. patient stratification for the VOYAGE 1 study.** ADA, adalimumab; GUS, guselkumab; LS, lesional skin; NL, nonlesional skin; PBO, placebo.

At week 0, 837 eligible participants were randomized (2:1:2) to 1 of 3 treatment arms: group I (n = 329, 100 mg guselkumab at weeks 0, 4, and 12 and every 8 weeks thereafter through week 44); group II (n = 174, placebo at weeks 0, 4, and 12 and then 100 mg guselkumab at weeks 16 and 20 and every 8 weeks thereafter through week 44); and group III (n = 334, 80 mg ADA at week 0, followed by 40 mg at week 1 and every 2 weeks thereafter through week 47). The open-label guselkumab treatment period began at week 48 and extended through week 264. Participants in all 3 treatment arms received 100 mg guselkumab at week 52 and every 8 weeks thereafter through week 252 (Figure 8).

Sterling Institutional Review Board was a central United States institutional review board for the VOYAGE 1 studies (4809C). Additional details can be found in Supplementary Table S1. Participants provided written informed consent before study initiation. Biomarker data were collected through week 48 of the study. Exploratory analyses were not based on a prespecified analysis plan.

### Biomarker assessments

#### Blood sample collection and analysis

Blood samples collected at weeks 0, 4, 24, and 48 were analyzed for the presence of serum-based biomarkers that have been reported to be differentially expressed between patients with psoriasis and healthy controls (ie, IL-17A, IL-17F, IL-22, and IL-23) as well as biomarkers associated with the mechanism of action of guselkumab in psoriasis (ie, CCL22/MDC, CCL4/MIP-1β, and CXCL/IL-8). A subset of randomly selected serum samples was analyzed using qualified antibody-based assays according to the manufacturer’s instructions. Specifically, IL-17A, IL-17F, IL-22, and IL-23 were measured with the Single Molecule Counting Erenna Assay; lower limits of quantification for IL-17A, IL-17F, IL-22, and IL-23 were 0.034, 0.39, 0.24, and 0.1 pg/ml, respectively. Additional biomarkers (CCL22/MDC, CCL4/MIP-1β, and CXCL/IL-8) were assessed using the Meso Scale Discovery immunoassay platform. To identify differences in baseline (week 0) serum profiles between the VOYAGE 1 psoriasis population and unaffected individuals, an independent, demographically matched healthy control cohort (n = 25 serum samples collected independent of the VOYAGE 1 study) (Table 7) was obtained commercially from Bioreclamation. Blood samples were analyzed at Janssen Research & Development, LLC.

**Table 7:** Matched Healthy Controls

NHC ID	Sex	Age	Race	BMI
NHC01	Male	49	White	32
NHC02	Male	60	Hispanic	22
NHC04	Male	40	White	24
NHC05	Male	51	White	28
NHC06	Male	61	White	32
NHC07	Male	60	White	28
NHC08	Male	49	White	22
NHC09	Male	48	White	21
NHC10	Female	58	White	30
NHC11	Female	43	White	25
NHC12	Female	62	White	29
NHC13	Female	55	White	22
NHC14	Female	43	White	35
NHC15	Female	42	Black	44
NHC16	Female	57	Black	19
NHC17	Female	40	Hispanic	41
NHC18	Female	60	White	33
NHC19	Female	44	Black	30
NHC22	Female	48	White	24
NHC29	Female	51	White	
NHC30	Female	49	White	
NHC52	Female	55	White	29
NHC53	Female	58	White	32
NHC55	Female	40	White	30
NHC57	Female	58	White	37
Mean value		50.70883		28.43763

Abbreviation: NHC, normal healthy controls.

Demographics of healthy controls is shown. Serum was obtained commercially from Bioreclamation.

#### Skin sample collection and analysis

LS biopsies were isolated from a representative psoriatic target lesion (≥3 cm) located on the trunk or extremities with approximately ≥0.5 mm of induration and approximately 5 mm from the edge of the plaque. Four 4-mm skin biopsies were obtained from the identified target lesion at week 0 prior to injection of study agent and at weeks 4, 24, and 48 after treatment (1 biopsy per visit). One additional punch biopsy was obtained at week 0 from uninvolved (ie, macroscopically normal or NL) skin at a body site similar to that of the biopsied lesion. All skin samples were collected, snap frozen in liquid nitrogen, and stored at −80 °C until RNA extraction. RNA was extracted using QIAsymphony RNA Kit (Qiagen) and later hybridized to GeneChip HT HG-U133+ perfect match 96-Array Plate (Affymetrix).

#### qRT-PCR

Data from qRT-PCR were generated by BioProcessing Solutions (RUCDR Infinite Biologics) with a Fluidigm BioMARK system using a panel for 121 preselected genes. Raw data were processed using Fluidigm RT-PCR software. β-Actin and 18s assays were run as controls on each array and for each gene/target. Delta Ct was calculated using β-actin as normalizer (reference control). Data quality control was performed to check the reproducibility among 3 replicates and the number of missing (undetermined) values. Six genes (*IL23A*, *IL12p35*, *TNF*, *IFGg*, *DEFB4*, and *CXCL10*) profiled using Fluidigm RT-PCR panel plus 3 rerun genes (*IL17A*, *IL17F*, and *IL22*) were included in the Figure 7 plot because of their direct relevance to IL-17/IL-23 pathway

The other rerun genes—*S100A7A*, *IL23R*, and *EBI3*—were not included in the plots owing to space limitations; however, their FCs are included in Table 8.

**Table 8:** *P-*Values and Multiple Comparison Corrected FDR

Cytokine	Visit	TRT	emmean	SE	df	lower.CL	upper.CL	Subject_Num	tScore	*P*-Value	FDR
IL-17A	wk4	Adalimumab	−0.59813	0.147615	112	−0.89061	−0.30565	37	−4.05199	9.40E-05	0.000174
IL-17A	wk4	Guselkumab	−1.00896	0.139897	112	−1.28615	−0.73177	40	−7.21215	6.93E-11	2.30E-10
IL-17A	wk4	Placebo to guselkumab	−0.21953	0.142896	112	−0.50266	0.063605	39	−1.53626	.13	0.16
IL-17A	wk24	Adalimumab	−1.29601	0.165337	105	−1.62385	−0.96818	34	−7.83859	3.94E-12	1.55E-11
IL-17A	wk24	Guselkumab	−1.70926	0.156396	105	−2.01937	−1.39916	37	−10.9291	5.05E-19	1.06E-17
IL-17A	wk24	Placebo to guselkumab	−1.48229	0.155435	105	−1.79049	−1.17409	38	−9.5364	6.73E-16	5.30E-15
IL-17A	wk48	Adalimumab	−0.94491	0.21545	100	−1.37235	−0.51746	31	−4.38574	2.86E-05	5.64E-05
IL-17A	wk48	Guselkumab	−1.69498	0.196839	100	−2.0855	−1.30445	36	−8.61098	1.08E-13	5.69E-13
IL-17A	wk48	Placebo to guselkumab	−1.67378	0.195089	100	−2.06083	−1.28673	37	−8.57958	1.27E-13	6.14E-13
IL-17F	wk4	Adalimumab	−0.72682	0.144634	112	−1.0134	−0.44025	37	−5.02525	1.92E-06	3.90E-06
IL-17F	wk4	Guselkumab	−1.27187	0.139521	112	−1.54831	−0.99543	40	−9.11598	3.62E-15	2.53E-14
IL-17F	wk4	Placebo to guselkumab	−0.28974	0.141506	112	−0.57012	−0.00937	39	−2.04757	.042942	0.059872
IL-17F	wk24	Adalimumab	−1.30587	0.202579	105	−1.70755	−0.9042	34	−6.44623	3.56E-09	8.63E-09
IL-17F	wk24	Guselkumab	−1.92576	0.194646	105	−2.3117	−1.53981	37	−9.89364	1.06E-16	1.12E-15
IL-17F	wk24	Placebo to guselkumab	−1.65035	0.192219	105	−2.03149	−1.26922	38	−8.58582	8.93E-14	5.63E-13
IL-17F	wk48	Adalimumab	−1.37161	0.20682	100	−1.78193	−0.96128	31	−6.63188	1.71E-09	4.50E-09
IL-17F	wk48	Guselkumab	−2.09804	0.191694	100	−2.47836	−1.71772	36	−10.9447	8.49E-19	1.34E-17
IL-17F	wk48	Placebo to guselkumab	−2.10672	0.18954	100	−2.48276	−1.73068	37	−11.1149	3.62E-19	1.06E-17
IL-22	wk4	Adalimumab	−0.42821	0.120403	112	−0.66677	−0.18965	37	−3.55648	.000552	0.00094
IL-22	wk4	Guselkumab	−0.77461	0.11536	112	−1.00319	−0.54604	40	−6.71473	8.14E-10	2.23E-09
IL-22	wk4	Placebo to guselkumab	−0.10187	0.116317	112	−0.33233	0.1286	39	−0.87577	.38	0.40
IL-22	wk24	Adalimumab	−0.66416	0.155116	104	−0.97176	−0.35656	34	−4.28171	4.14E-05	7.91E-05
IL-22	wk24	Guselkumab	−1.07006	0.150568	104	−1.36865	−0.77148	36	−7.10683	1.54E-10	4.62E-10
IL-22	wk24	Placebo to guselkumab	−0.77268	0.146153	104	−1.0625	−0.48285	38	−5.28675	6.91E-07	1.56E-06
IL-22	wk48	Adalimumab	−0.63489	0.158568	100	−0.94949	−0.3203	31	−4.00392	.00012	0.000216
IL-22	wk48	Guselkumab	−1.21593	0.146936	100	−1.50745	−0.92442	36	−8.27526	5.79E-13	2.61E-12
IL-22	wk48	Placebo to guselkumab	−1.08685	0.144044	100	−1.37263	−0.80107	37	−7.54524	2.13E-11	7.90E-11
IL-23	wk4	Adalimumab	−0.26747	0.053136	110	−0.37277	−0.16217	36	−5.03373	1.89E-06	3.90E-06
IL-23	wk4	Guselkumab	−0.04884	0.050481	110	−0.14889	0.051197	40	−0.96758	.34	0.36
IL-23	wk4	Placebo to guselkumab	−0.10608	0.051994	110	−0.20912	−0.00304	38	−2.04029	.043716	0.059872
IL-23	wk24	Adalimumab	−0.1435	0.06541	101	−0.27325	−0.01374	33	−2.1938	.030543	0.043732
IL-23	wk24	Guselkumab	−0.01792	0.062609	101	−0.14212	0.106279	36	−0.28623	.78	0.79
IL-23	wk24	Placebo to guselkumab	−0.10088	0.06283	101	−0.22552	0.023756	36	−1.60563	.11	0.14
IL-23	wk48	Adalimumab	−0.20941	0.056757	96	−0.32207	−0.09674	29	−3.68953	.000373	0.000652
IL-23	wk48	Guselkumab	−0.04847	0.050914	96	−0.14954	0.052589	36	−0.95208	.34	0.36
IL-23	wk48	Placebo to guselkumab	−0.06347	0.051897	96	−0.16648	0.039549	35	−1.22292	.22	0.26
IL-8	wk4	Adalimumab	−0.20747	0.124402	108	−0.45405	0.039118	35	−1.66773	.10	0.13
IL-8	wk4	Guselkumab	−0.16878	0.121137	108	−0.40889	0.071339	38	−1.39326	.17	0.20
IL-8	wk4	Placebo to guselkumab	−0.1933	0.118895	108	−0.42897	0.042375	39	−1.62577	.11	0.14
IL-8	wk24	Adalimumab	−0.28877	0.1062	102	−0.49942	−0.07812	33	−2.71911	.007695	0.012119
IL-8	wk24	Guselkumab	−0.23708	0.103123	102	−0.44163	−0.03254	36	−2.29902	.023543	0.034493
IL-8	wk24	Placebo to guselkumab	−0.34386	0.100966	102	−0.54412	−0.1436	37	−3.40572	.000945	0.001567
IL-8	wk48	Adalimumab	−0.25963	0.106352	98	−0.47068	−0.04858	30	−2.44122	.016432	0.024647
IL-8	wk48	Guselkumab	−0.27151	0.100527	98	−0.47101	−0.07202	35	−2.70091	.008148	0.012521
IL-8	wk48	Placebo to guselkumab	−0.18253	0.096071	98	−0.37318	0.008117	37	−1.89997	.060376	0.08093
MDC	wk4	Adalimumab	−0.49239	0.068861	106	−0.62891	−0.35587	34	−7.15053	1.16E-10	3.65E-10
MDC	wk4	Guselkumab	−0.34511	0.065772	106	−0.47551	−0.21471	37	−5.24707	7.98E-07	1.73E-06
MDC	wk4	Placebo to guselkumab	−0.07272	0.064439	106	−0.20048	0.055033	39	−1.12856	.26	0.30
MDC	wk24	Adalimumab	−0.62614	0.083811	101	−0.7924	−0.45988	32	−7.47088	2.93E-11	1.03E-10
MDC	wk24	Guselkumab	−0.50527	0.078618	101	−0.66123	−0.34932	36	−6.42692	4.35E-09	1.01E-08
MDC	wk24	Placebo to guselkumab	−0.50814	0.077849	101	−0.66257	−0.35371	37	−6.52731	2.72E-09	6.85E-09
MDC	wk48	Adalimumab	−0.74717	0.086068	96	−0.91801	−0.57633	30	−8.68113	1.01E-13	5.69E-13
MDC	wk48	Guselkumab	−0.56404	0.080232	96	−0.7233	−0.40478	34	−7.03012	3.01E-10	8.62E-10
MDC	wk48	Placebo to guselkumab	−0.62944	0.078315	96	−0.78489	−0.47399	36	−8.03731	2.37E-12	9.95E-12
MIP1β	wk4	Adalimumab	−0.70247	0.045008	106	−0.79171	−0.61324	34	−15.6079	3.14E-29	1.98E-27
MIP1β	wk4	Guselkumab	0.008844	0.043151	106	−0.07671	0.094396	37	0.204955	.84	0.84
MIP1β	wk4	Placebo to guselkumab	−0.05653	0.042029	106	−0.13985	0.026801	39	−1.34492	.18	0.22
MIP1β	wk24	Adalimumab	−0.52527	0.053783	101	−0.63196	−0.41857	32	−9.76631	2.94E-16	2.64E-15
MIP1β	wk24	Guselkumab	−0.05264	0.050679	101	−0.15317	0.047894	36	−1.03868	.30	0.33
MIP1β	wk24	Placebo to guselkumab	−0.15329	0.05008	101	−0.25264	−0.05395	37	−3.06097	.002827	0.004566
MIP1β	wk48	Adalimumab	−0.57141	0.053382	96	−0.67738	−0.46545	30	−10.7042	4.61E-18	5.80E-17
MIP1β	wk48	Guselkumab	−0.05385	0.050201	96	−0.1535	0.045795	34	−1.07274	.29	0.32
MIP1β	wk48	Placebo to guselkumab	−0.05105	0.04879	96	−0.14789	0.0458	36	−1.04627	.30	0.33

Abbreviations: df, degree of freedom; FDR, false discovery rate; MDC, macrophage-derived chemokine; SE, standard error; TRT, treatment.

Emmean denotes linear regression model derived expected least square mean.

### Statistical analyses

#### Serum biomarker analysis

Welch’s *t*-test was applied for each individual biomarker, in log_2_-transformed concentrations, between 118 patients with psoriasis at baseline (week 0) and 25 healthy controls randomly selected according to the age, sex, race, and body mass index distribution of the psoriasis population (Table 7) (samples collected from Bioreclamation) to assess for differences in baseline biomarker profiles between the study psoriasis population and healthy controls; an ANOVA was applied to evaluate for differences in baseline biomarker profiles among participants with psoriasis assigned to the 3 treatment arms (guselkumab, ADA, and placebo). PD effects in response to individual treatment were determined at weeks 4, 24, and 48 against baseline (week 0), using analysis of covariance linear mixed-effects models (lme) in R (https://cran.r-project.org/web/packages/emmeans/index.html) for each log_2_ concentration by including treatment group, time, time by group interaction, and baseline log2 concentration as fixed effects and patient as a random effect.

Adjustment for multiple pairwise comparisons was implemented using Benjamini–Hochberg FDR to account to account for multiple comparisons to baselines at different time points. The summary table of the *P*-values and the multiple comparison–corrected FDR are attached in Table 8.

#### Skin transcriptomics analysis

Gene expression measures in individual skin biopsies were determined at the probe set level (n = 54,715) using the Robust Multi-array Average methodology. The quality of the entire dataset (183 skin samples) was assessed using a principal component analysis of whole-genome expression profiling, and no apparent outlier samples were detected.

Differential gene expression between psoriatic LS and NL at baseline (week 0) was evaluated using a paired *t*-test among 37 patients. *P*-values from paired *t*-tests were adjusted for multiple hypotheses using the Benjamini–Hochberg FDR procedure. A FC >1.5 and FDR <0.05 were employed as criteria to determine the PSTR at baseline. For each probe set (or gene) in the PSTR, percentage of improvement in expression in LS in response to individual treatment at a given time point was calculated as the negative ratio of the average of the log_2_ ratio of treatment (LS after treatment vs LS at baseline) over the average of the log_2_ ratio of disease profiling (LS vs NL at baseline) among all patients in the given treatment arm. The molecular scar was defined for each treatment as the subset of PSTR genes with <75% improvement and remaining FC >1.5 in healed LS collected from patients when they achieved PASI100 response.

Canonical pathways in the QIAGEN Ingenuity Pathway Analysis software (QIAGEN, https://digitalinsights.qiagen.com/IPA) enriched with PSTR genes were identified by a hypergeometric test (Krämer et al, 2014). A total of 56 pathways were selected with *P* < .01 and having >20% and ≥5 pathway genes as PSTR genes. For each of the 56 pathways, the percentage of improvement in expression in LS in response to individual treatments at a given time point was calculated as the average of the percentage of improvement among all PSTR genes in the pathway, with associated standard error.

Separately, the overall differential expression in 124 curated gene sets related to immune cell and skin inflammation between LS and NL at baseline was evaluated by GSVA. A total of 108 gene sets exhibited significant differences in GSVA scores between LS and NL at baseline (FDR < 0.05). Overall, 71 of these 108 gene sets were enriched with PSTR genes (FDR < 0.05, having >20% and ≥5 genes in each gene set as PSTR genes). For each of the 71 gene sets, percentage of improvement in expression in LS in response to individual treatment at a given time point was calculated as the average of percentage of improvement among all PSTR genes in the pathway, with associated standard error.

### Clinical trial information

The primary results of this study have been published with the ClinicalTrials.gov identifier NCT02207231 and can be found in the abstract and Materials and Methods: ClinicalTrials.gov identifier NCT02207231 for the abstract and NCT02207231 for the Materials and Methods. Further identifying details have been added to the Material and Methods. Blood sample collection and analysis were performed at Janssen Research & Development, LLC.

## Ethics Statement

Sterling Institutional Review Board was a central United States institutional review board for the VOYAGE 1 studies (4809C). Additional details can be found in Supplementary Table S6. Patients provided written informed consent before study initiation. The primary results of this study, including additional details on approvals, have been published (Blauvelt et al, 2017, Supplementary Table S1).

## Data Availability Statement

Raw data have been deposited in the National Center for Biotechnology Information’s Gene Expression Omnibus with accession number GSE252029: https://www.ncbi.nlm.nih.gov/geo/query/acc.cgi?acc=GSE252029.

## ORCIDs

Andrew Blauvelt: http://orcid.org/0000-0002-2633-985X

Richard G. Langley: http://orcid.org/0000-0003-4730-2164

Patrick J. Branigan: http://orcid.org/0000-0001-9741-9067

Xuejun Liu: http://orcid.org/0000-0001-8194-3067

Yanqing Chen: http://orcid.org/0000-0002-9534-6199

Samuel DePrimo: http://orcid.org/0000-0002-4195-8616

Keying Ma: http://orcid.org/0000-0003-3454-9168

Brittney Scott: http://orcid.org/0000-0003-4647-5303

Kim Campbell: http://orcid.org/0000-0003-3789-416X

Ernesto J. Muñoz-Elías: http://orcid.org/0000-0002-2894-157X

Kim A. Papp: http://orcid.org/0000-0001-9557-3642

## Conflict of Interest

AB has served as a scientific advisor, clinical study investigator, and speaker for AbbVie and as a scientific advisor and/or clinical study investigator for Abcentra, Aligos, Almirall, Amgen, Arcutis, Arena, Aslan, Athenex, Boehringer Ingelheim, Bristol Myers Squibb, Celgene, Dermavant, EcoR1, Eli Lilly, Evommune, Forte, Galderma, Incyte, Janssen, Landos, Leo, Novartis, Pfizer, Rapt, Regeneron, Sanofi Genzyme, Sun Pharma, UCB Pharma, and Vibliome. RGL has been a principal investigator, served on an advisory board, or served as a speaker for AbbVie, Amgen, Boehringer Ingelheim, Celgene, Eli Lilly, Janssen, Leo Pharma, Merck, Novartis, Pfizer, Sun Pharma, and UCB Pharma. KAP has received clinical research grants from; has received honoraria from; and/or has served as a consultant, scientific advisor, investigator, consultant, speaker, and/or medical officer for AbbVie, Akros, Amgen, Anacor, Arcutis, Astellas, Avillion, Bausch Health, Baxalta, Boehringer Ingelheim, Bristol Myers Squibb, Can-Fite Biopharma, Celgene, Celltrion, Coherus, Dermavant, Dermira, Dice Pharmaceuticals, Dow Pharma, Eli Lilly, Evelo, Galapagos, Galderma, Genentech, Gilead, GlaxoSmithKline, Incyte, Janssen, Kyowa Hakko Kirin, LEO Pharma, Medimmune, Meiji Seika Pharma, Merck Sharpe & Dohme, Merck-Serono, Mitsubishi Pharma, Moberg Pharma, Novartis, Pfizer, PRCL Research, Regeneron, Reistone, Roche, Sandoz, Sanofi-Aventis/Genzyme, Sun Pharma, Takeda, UCB Pharma, Valean, vTv Therapeutics, and Xencor. XL, SD, and EJM-E are former employees of Janssen. PJB, YC, KM, BS, and KC are employees of Janssen and own stock in Johnson and Johnson.

## Figures and Tables

**Table S1:** List of Institutional Review Boards and Ethics Committees

Region	IRB/EC Name/Address	IRB/EC Chairperson	Site	Approval Date
Australia	BELLBERRY LTD 129 Glen Osmond Road N/A N/A Eastwood, SA, 5063 Australia		AU00211 1/80 Drummond Street Carlton, Victoria, 3053 Australia Received drug	Region: Nov 17, 2014 Site, Central: Nov 17, 2014 Site, Local: /
Australia	BELLBERRY LTD 129 Glen Osmond Road N/A N/A Eastwood, SA, 5063 Australia		AU00213 3 Balaclava Street Woolloongabba, QLD, 4102 Australia Received drug	Region: Nov 17, 2014 Site, Central: Nov 17, 2014 Site, Local: /
Australia	BELLBERRY LTD 129 Glen Osmond Road N/A N/A Eastwood, SA, 5063 Australia		AU00217 121 Crown Street Sydney, NSW, 2010 Australia Received drug	Region: Nov 17, 2014 Site, Central: Nov 17, 2014 Site, Local: /
Australia	Western Sydney Local Health District Cnr Hawkesbury Rd and Darcy Rd Westmead, NSW, 2145 Australia		AU00218 Cnr Hawkesbury And Darcy Rd Sydney, NSW, 2145 Australia Received drug	Region: Nov 17, 2014 Site, Central: / Site, Local: Mar 26, 2015
Australia	BELLBERRY LTD 129 Glen Osmond Road N/A N/A Eastwood, SA, 5063 Australia		AU00219 230, St Bernards Road Hectorville, South Australia, 5073 Australia Received drug	Region: Nov 17, 2014 Site, Central: Nov 17, 2014 Site, Local: /
Australia	BELLBERRY LTD 129 Glen Osmond Road N/A N/A Eastwood, SA, 5063 Australia		AU00220 Level 1, 1 Bowes Place, PHILLIP Woden, Australia Capital Territory, 2606 Australia Received drug	Region: Nov 17, 2014 Site, Central: Nov 17, 2014 Site, Local: /
Australia	BELLBERRY LTD 129 Glen Osmond Road N/A N/A Eastwood, SA, 5063 Australia		AU00254 SINCLAIR Dermatology EAST MELBOURNE, VICTORIA, 3002 Australia Received drug	Region: Nov 17, 2014 Site, Central: Nov 17, 2014 Site, Local: /
Australia	BELLBERRY LTD 129 Glen Osmond Road N/A N/A Eastwood, SA, 5063 Australia		AU00255 1/ 7 Secant Street Liverpool, NSW, 2170 Australia Received drug	Region: Nov 17, 2014 Site, Central: Nov 17, 2014 Site, Local: /
Canada	Health Research Ethics Authority 95 Bonaventure Ave, Suite 200 Eastern Trust Bldg St.John's, NL, A1B 2X5 Canada		CA00244 120 Stavanger Drive, Suite 102 St. John's, NL, A1B 3E1 Canada Received drug	Region: Oct 17, 2014 Site, Central: / Site, Local: Dec 22, 2014
Canada	Institutional Review Board Services 372 Hollandview Trail Suite 300 Aurora, ON, L4G 0A5 Canada		CA00246 743 Lansdowne St W Peterborough, ON, K9J 1Z2 Canada Received drug	Region: Oct 17, 2014 Site, Central: Nov 25, 2014 Site, Local: /
Canada	Institutional Review Board Services 372 Hollandview Trail Suite 300 Aurora, ON, L4G 0A5 Canada		CA00247 1851 Sherbrooke Street East Montreal, QC, H2K4L5 Canada Received drug	Region: Oct 17, 2014 Site, Central: Oct 17, 2014 Site, Local: /
Canada	Institutional Review Board Services 372 Hollandview Trail Suite 300 Aurora, ON, L4G 0A5 Canada		CA00250 500 Cassells St North Bay, ON, P1B 3Z7 Canada Received drug	Region: Oct 17, 2014 Site, Central: Nov 25, 2014 Site, Local: /
Canada	Health Research Ethics Board of Alberta Cancer Committee 1500, 10104 - 103 Avenue NW Edmonton, Alberta, T5J 4A7 Canada		CA00253 10140 117 St Nw Edmonton, AB, T5K 1X3 Canada Received drug	Region: Oct 17, 2014 Site, Central: / Site, Local: Jan 06, 2015
Canada	Health Research Ethics Board of Alberta Cancer Committee 1500, 10104 - 103 Avenue NW Edmonton, Alberta, T5J 4A7 Canada		CA00254 600 Crowfoot Crescent Northwest Calgary, AB, T3G 0B4 Canada Received drug	Region: Oct 17, 2014 Site, Central: / Site, Local: Feb 03, 2015
Canada	Institutional Review Board Services 372 Hollandview Trail Suite 300 Aurora, ON, L4G 0A5 Canada		CA00281 95 Bayly Street West Ajax, ON, L1S 7K8 Canada Received drug	Region: Oct 17, 2014 Site, Central: Nov 27, 2014 Site, Local: /
Canada	Institutional Review Board Services 372 Hollandview Trail Suite 300 Aurora, ON, L4G 0A5 Canada		CA00289 Siena Medical Research Corporation Montreal, QC, H3Z 2S6 Canada Received drug	Region: Oct 17, 2014 Site, Central: Nov 25, 2014 Site, Local: /
Canada	Captial Health Research Ethics Board Centre for Clinical Research 5790 University Avenue Room 118 Halifax, NS, B3H 1V7 Canada		CA90077 6054 Coburg Road Halifax, NS, B3H 1Z2 Canada Received drug	Region: Oct 17, 2014 Site, Central: / Site, Local: Mar 09, 2015
Canada	UBC Office of Research Ethics - CREB Room 210 828 West 10th Avenue Vancouver, BC, V5Z 1L8 Canada		CA90165 835 West 10th Avenue Vancouver, BC, V5Z 4E8 Canada Received drug	Region: Oct 17, 2014 Site, Central: / Site, Local: Dec 10, 2014
Canada	Institutional Review Board Services 372 Hollandview Trail Suite 300 Aurora, ON, L4G 0A5 Canada		CA90202 835 Richmond St London, ON, N6A 3H7 Canada Received drug	Region: Oct 17, 2014 Site, Central: Nov 25, 2014 Site, Local: /
Germany	Landesamt für Gesundheit und Soziales Berlin Geschaeftsstelle der Ethik-Kommission des Landes Berlin Fehrbellinerplatz 1 LaGeSo Berlin Geschäftsstelle der EK des Landes Berlin Berlin, 10707 Germany	Moesta Thomas	DE00292 Chariteplatz 1 Berlin, 10117 Germany Received drug	Region: Dec 17, 2014 Site, Central: Dec 17, 2014 Site, Local: Dec 17, 2014
Germany	ETHIK KOMMISSION DER LANDESÄRZTEKAMMER BRANDENBURG - HAUPTGESCHÄFTSSTELLE Dreifertstr 12 Cottbus, 03044 Germany	MATTHIAS MICHAEL	DE00480 Am Bahnhof 1 Mahlow, 15831 Germany Received drug	Region: Dec 17, 2014 Site, Central: Dec 17, 2014 Site, Local: Dec 17, 2014
Germany	ETHIKKOMMISSION LÄK THÜRINGEN Im Semmicht 33 Ethik-Kommission JENA, 07751 Germany	Brandl Ulrich	DE00481 Straße des Friedens 122 Gera, 07548 Germany Received drug	Region: Dec 17, 2014 Site, Central: Dec 17, 2014 Site, Local: Dec 17, 2014
Germany	Ethik-Kommission der Med. Fak. der Eberhard-Karls-Universität und am Universitätsklinikum Tübingen Gartenstr 47 Tübingen, 72074 Germany	Luft Dieter	DE00482 Liebermeisterstr 25 Tübingen, 72076 Germany Received drug	Region: Dec 17, 2014 Site, Central: Dec 17, 2014 Site, Local: Dec 17, 2014
Germany	ETHIKKOMMISSION DER UNIVERSITÄT DUISBURG-ESSEN Robert-Koch-Str. 9-11 Ethik-Kommission der Med. Fakultät d. Universität Essen, 4514 Germany	Havers Werner	DE00483 Hufelandstrasse 55 ESSEN, NORDRHEIN-WESTFALEN, 45122 Germany Received drug	Region: Dec 17, 2014 Site, Central: Nov 05, 2014 Site, Local: Nov 05, 2014
Germany	ETHIK KOMMISSION DER RHEINISCHE FRIEDRICH-WILHELMS UNIVERSITÄT BONN Biomedizinisches Zentrum, Sigmund-Freud-Strasse 25 Bonn, 53105 Germany	Racké K.	DE00485 Sigmund-Freud-Straße 25 BONN, NORDRHEIN-WESTFALEN, 53105 Germany Received drug	Region: Dec 17, 2014 Site, Central: Dec 17, 2014 Site, Local: Dec 17, 2014
Germany	Ethik-Kommission der Medizinischen Fakultaet Carl Gustav Carus der TU Dresden Fetscherstraße 74 Dresden, 01307 Germany	Platzbecker Uwe	DE00486 Fetscher Str. 74 Dresden, 01307 Germany Received drug	Region: Dec 17, 2014 Site, Central: Dec 17, 2014 Site, Local: Dec 17, 2014
Germany	ETHIKKOMMISSION DER MEDIZINISCHEN FAKULTAT DER CHRISTIAN-ALBRECHTS-UNIVERSITAT ZU KIEL SCHWANENWEG 20 KIEL, 24105 Germany	Mehdorn Maximilian	DE00488 Schittenhelmstrasse 7 Kiel, 24105 Germany Received drug	Region: Dec 17, 2014 Site, Central: Dec 17, 2014 Site, Local: Dec 17, 2014
Germany	Ethikkommission der Sächsischen Landesärztekammer Schützenhöhe 16-18 Dresden, 01099 Germany	Terhaag Bernd	DE00490 Prager Str. 10 Dresden, Sachsen, 01069 Germany Received drug	Region: Dec 17, 2014 Site, Central: Mar 05, 2015 Site, Local: Mar 05, 2015
Germany	Ethikkommission der Medizinischen Fakultät der Westfälischen Wilhelms-Universität Münster und der Gartenstr. 210-214 MUENSTER, 48147 Germany	Bothe Hans-Werner	DE00491 Von-Esmarch-Strabe 58 Munster, 48149 Germany Received drug	Region: Dec 17, 2014 Site, Central: Oct 29, 2014 Site, Local: Oct 29, 2014
Germany	Ethikkommission und klinisches Ethik-Kommittee der Medizinischen Fakultaet der Universitaet Luebeck Ratzeburger Allee 160 UKSH Campus Lübeck Luebeck, Schleswig-Holstein, 23538 Germany	Katalinic A., M.D.	DE00492 Ratzeburger Allee 160 LÜBECK, Schlesw.-H, 23538 Germany Received drug	Region: Dec 17, 2014 Site, Central: Jan 29, 2015 Site, Local: Jan 29, 2015
Germany	Landesamt für Gesundheit und Soziales Berlin Geschaeftsstelle der Ethik-Kommission des Landes Berlin Fehrbellinerplatz 1 LaGeSo Berlin Geschäftsstelle der EK des Landes Berlin Berlin, 10707 Germany	Moesta Thomas	DE00493 Rankestr. 34 Berlin, 10789 Germany Received drug	Region: Dec 17, 2014 Site, Central: Dec 17, 2014 Site, Local: Dec 17, 2014
Germany	Ethikkommission der Aerztekammer Hamburg Weidestr. 122b HAMBURG, 22083 Germany	STAHL ROLF	DE00495 Drehbahn 1-3 Hamburg, 20354 Germany Received drug	Region: Dec 17, 2014 Site, Central: Jan 29, 2015 Site, Local: Jan 29, 2015
Germany	Ethik-Kommission des Fachbereichs Medizin der Johann-Wolfgang-Goethe Universität Theodor-Stern-Kai 7 HAUS 1 Frankfurt, 60590 Germany	Harder Sebastian	DE00496 Klinik für Dermatologie, Venerologie und Allergologie Franfurt, 60590 Germany Received drug	Region: Dec 17, 2014 Site, Central: Mar 11, 2015 Site, Local: Mar 11, 2015
Hungary	Medical Research Council Arany Janos u. 6-8 Budapest, 1051 Hungary	FÜRST ZSUZSANNA, M.D.	HU36001 Nagyerdei Körút 98 Debrecen, Hajdú-Bihar Megye, 4032 Hungary Received drug	Region: Nov 14, 2014 Site, Central: Nov 14, 2014 Site, Local: Nov 14, 2014
Hungary	Medical Research Council Arany Janos u. 6-8 Budapest, 1051 Hungary	FÜRST ZSUZSANNA, M.D.	HU36002 Korányi fasor 6. Szeged, Csongrád, 6720 Hungary Received drug	Region: Nov 14, 2014 Site, Central: Nov 14, 2014 Site, Local: Nov 14, 2014
Hungary	Medical Research Council Arany Janos u. 6-8 Budapest, 1051 Hungary	FÜRST ZSUZSANNA, M.D.	HU36003 Akác u. 1. PÉCS, Baranya, 7632 Hungary Received drug	Region: Nov 14, 2014 Site, Central: Nov 14, 2014 Site, Local: Nov 14, 2014
Hungary	Medical Research Council Arany Janos u. 6-8 Budapest, 1051 Hungary	FÜRST ZSUZSANNA, M.D.	HU36004 Mária u. 41 Budapest, 1085 Hungary Received drug	Region: Nov 14, 2014 Site, Central: Nov 14, 2014 Site, Local: Nov 14, 2014
Hungary	Medical Research Council Arany Janos u. 6-8 Budapest, 1051 Hungary	FÜRST ZSUZSANNA, M.D.	HU36005 Tallián Gy. u. 20-32. Kaposvár, 7400 Hungary Received drug	Region: Nov 14, 2014 Site, Central: Nov 14, 2014 Site, Local: Nov 14, 2014
Hungary	Medical Research Council Arany Janos u. 6-8 Budapest, 1051 Hungary	FÜRST ZSUZSANNA, M.D.	HU36006 Nyíri út 38 Kecskemét, Bács-Kiskun Megye, 6000 Hungary Received drug	Region: Nov 14, 2014 Site, Central: Nov 14, 2014 Site, Local: Nov 14, 2014
Korea	Seoul National university Bundang Hospital IRB 82, Gumi-ro 173 Beon-gil, Bundang-gu Seongnam, Gyeonggi-do, 463-707 Korea		KR00155 82 Seongnam-Si, Gyeonggi-do, 463-707 Korea Received drug	Region: Sep 18, 2014 Site, Central: / Site, Local: Oct 13, 2014
Korea	Samsung Medical Center Institutional Review Board 50 Ilwondong, Kangnam-gu, Seoul Seoul, 135-710 Korea		KR00156 81, Irwon-ro, Gangnam-gu Seoul, 135-710 Korea Received drug	Region: Sep 18, 2014 Site, Central: / Site, Local: Oct 17, 2014
Korea	Chungnam National University hospital Chungnam National University Hospital 640 Daesa-dong, Joong-gu Daejeon, 301-721 Korea		KR00157 282 Munhwa-ro Jung-gu Daejeon, 301-721 Korea Received drug	Region: Sep 18, 2014 Site, Central: / Site, Local: Nov 19, 2014
Korea	The Catholic University of Korea Seoul St. Mary’s Hospital 222 Banpo-daero, Seocho-gu #202 8F, The Catholic University of Korea Seoul St. Mary’s Hospital Seoul, 137-701 Korea		KR00158 222 Banpo-daero, Seocho-gu Seoul, 137-701 Korea Received drug	Region: Sep 18, 2014 Site, Central: / Site, Local: Oct 07, 2014
Korea	Pusan National University Hospital IRB Pusan National University Hospital 179 Gudeok-ro, Seo-Gu Busan, 602-739 Korea		KR00160 179 Gudeok-Ro, Seo-Gu Busan, 602-739 Korea Received drug	Region: Sep 18, 2014 Site, Central: / Site, Local: Oct 11, 2014
Korea	Ajou University Hospital IRB Ajou University Hospital, San 5, Wonchon-dong, Yeongdong-gu Suwon, Gyeonggi-do, 443-721 Korea		KR00161 164 Worldcup-ro Yeongtong-gu Suwon, Gyeonggi-do, 443-380 Korea Received drug	Region: Sep 18, 2014 Site, Central: / Site, Local: Sep 18, 2014
Poland	Komisja Bioetyczna przy Wydziale Nauk Medycznych Uniwersytetu Warminsko Mazurskiego Al. Warszawska 30 Olsztyn, 10-082 Poland		PL00227 Sikorskiego 13/U1 Warszawa, 02-758 Poland Received drug	Region: Sep 24, 2014 Site, Central: Sep 24, 2014 Site, Local: Sep 24, 2014
Poland	Komisja Bioetyczna przy Wydziale Nauk Medycznych Uniwersytetu Warminsko Mazurskiego Al. Warszawska 30 Olsztyn, 10-082 Poland		PL00230 Sklodowskiej-Curie 9, Bydgoszcz, 85-094 Poland Received drug	Region: Sep 24, 2014 Site, Central: Sep 24, 2014 Site, Local: Sep 24, 2014
Poland	Komisja Bioetyczna przy Wydziale Nauk Medycznych Uniwersytetu Warminsko Mazurskiego Al. Warszawska 30 Olsztyn, 10-082 Poland		PL00233 Wiejska 81 Bialystok, Podlaskie, 15-351 Poland Received drug	Region: Sep 24, 2014 Site, Central: Sep 24, 2014 Site, Local: Sep 24, 2014
Poland	Komisja Bioetyczna przy Wydziale Nauk Medycznych Uniwersytetu Warminsko Mazurskiego Al. Warszawska 30 Olsztyn, 10-082 Poland		PL00235 ul. Piotrkowska 48 Łódź, 90-265 Poland Received drug	Region: Sep 24, 2014 Site, Central: Sep 24, 2014 Site, Local: Sep 24, 2014
Poland	Komisja Bioetyczna przy Wydziale Nauk Medycznych Uniwersytetu Warminsko Mazurskiego Al. Warszawska 30 Olsztyn, 10-082 Poland		PL00240 Kosciuszki 93 Lodz, 90-436 Poland Received drug	Region: Sep 24, 2014 Site, Central: Sep 24, 2014 Site, Local: Sep 24, 2014
Poland	Komisja Bioetyczna przy Wydziale Nauk Medycznych Uniwersytetu Warminsko Mazurskiego Al. Warszawska 30 Olsztyn, 10-082 Poland		PL00246 Ul Chalubinskiego 1 Wroclaw N/a, 50-981 Poland Received drug	Region: Sep 24, 2014 Site, Central: Sep 24, 2014 Site, Local: Sep 24, 2014
Poland	Komisja Bioetyczna przy Wydziale Nauk Medycznych Uniwersytetu Warminsko Mazurskiego Al. Warszawska 30 Olsztyn, 10-082 Poland		PL00248 Al.Wojska Polskie Olsztyn, 11-041 Poland Received drug	Region: Sep 24, 2014 Site, Central: Sep 24, 2014 Site, Local: Sep 24, 2014
Russia	Independent Interdisciplinary Committee for Ethics Expertise of Clinical Trials 51 Leningradsky prospect Moscow, 125468 Russia		RU00366 32, Maksima Gorkogo Ul. Bataysk, 346882 Russia Received drug	Region: Sep 29, 2014 Site, Central: Sep 29, 2014 Site, Local: /
Russia	Independent Interdisciplinary Committee for Ethics Expertise of Clinical Trials 51 Leningradsky prospect Moscow, 125468 Russia		RU00367 42, Serebrennikovskaya str. Novosibirsk, 630099 Russia Did not receive drug	Region: Sep 29, 2014 Site, Central: Sep 29, 2014 Site, Local: /
Russia	Independent Interdisciplinary Committee for Ethics Expertise of Clinical Trials 51 Leningradsky prospect Moscow, 125468 Russia		RU00368 17, Leninsky pr. Moscow, 119071 Russia Did not receive drug	Region: Sep 29, 2014 Site, Central: Sep 29, 2014 Site, Local: /
Russia	Independent Interdisciplinary Committee for Ethics Expertise of Clinical Trials 51 Leningradsky prospect Moscow, 125468 Russia		RU00369 17, Leninsky pr. Moscow, 119071 Russia Received drug	Region: Sep 29, 2014 Site, Central: Sep 29, 2014 Site, Local: /
Russia	Independent Interdisciplinary Committee for Ethics Expertise of Clinical Trials 51 Leningradsky prospect Moscow, 125468 Russia		RU00370 9, Sportivnaya str. Ryazan, 390046 Russia Received drug	Region: Sep 29, 2014 Site, Central: Sep 29, 2014 Site, Local: /
Russia	Independent Interdisciplinary Committee for Ethics Expertise of Clinical Trials 51 Leningradsky prospect Moscow, 125468 Russia		RU00371 1K, Makarova str. Lipetsk, 398005 Russia Received drug	Region: Sep 29, 2014 Site, Central: Sep 29, 2014 Site, Local: /
Russia	Independent Interdisciplinary Committee for Ethics Expertise of Clinical Trials 51 Leningradsky prospect Moscow, 125468 Russia		RU00372 29A, Parkhomenko pr. St-Petersburg, 194021 Russia Received drug	Region: Sep 29, 2014 Site, Central: Sep 29, 2014 Site, Local: /
Russia	Independent Interdisciplinary Committee for Ethics Expertise of Clinical Trials 51 Leningradsky prospect Moscow, 125468 Russia		RU00373 Kirochnaya 41 St Petersburg, 191015 Russia Did not receive drug	Region: Sep 29, 2014 Site, Central: Sep 29, 2014 Site, Local: /
Russia	Independent Interdisciplinary Committee for Ethics Expertise of Clinical Trials 51 Leningradsky prospect Moscow, 125468 Russia		RU00374 112, Karla Marksa str. Kirov, 610027 Russia Received drug	Region: Sep 29, 2014 Site, Central: Sep 29, 2014 Site, Local: /
Russia	Independent Interdisciplinary Committee for Ethics Expertise of Clinical Trials 51 Leningradsky prospect Moscow, 125468 Russia		RU00375 20/1 Delegatskaya Str Moscow, 127473 Russia Received drug	Region: Sep 29, 2014 Site, Central: Sep 29, 2014 Site, Local: /
Russia	Independent Interdisciplinary Committee for Ethics Expertise of Clinical Trials 51 Leningradsky prospect Moscow, 125468 Russia		RU00382 31a, Sevryukova str. Stavropol, 355020 Russia Received drug	Region: Sep 29, 2014 Site, Central: Sep 29, 2014 Site, Local: /
Russia	Independent Interdisciplinary Committee for Ethics Expertise of Clinical Trials 51 Leningradsky prospect Moscow, 125468 Russia		RU90181 52, Dostoevskogo str. Kazan, 420097 Russia Did not receive drug	Region: Sep 29, 2014 Site, Central: Sep 29, 2014 Site, Local: /
Russia	Independent Interdisciplinary Committee for Ethics Expertise of Clinical Trials 51 Leningradsky prospect Moscow, 125468 Russia		RU90263 132, Dostoevsky str. Ufa, 450005 Russia Did not receive drug	Region: Sep 29, 2014 Site, Central: Sep 29, 2014 Site, Local: /
Russia	Independent Interdisciplinary Committee for Ethics Expertise of Clinical Trials 51 Leningradsky prospect Moscow, 125468 Russia		RU90279 8 Scherbakova Str Ekaterinburg, 620076 Russia Received drug	Region: Sep 29, 2014 Site, Central: Sep 29, 2014 Site, Local: /
Russia	Independent Interdisciplinary Committee for Ethics Expertise of Clinical Trials 51 Leningradsky prospect Moscow, 125468 Russia		RU90282 179 Rashpilevskaya str. Krasnodar, 350020 Russia Received drug	Region: Sep 29, 2014 Site, Central: Sep 29, 2014 Site, Local: /
Russia	LEC V A Nassonova Research Institute of Rheumatology Kashirskoye shosse, d. 34A Moscow, 115522 Russia		RU90288 34A, Krashirskoe Shosse Moscow, 115522 Russia Received drug	Region: Sep 29, 2014 Site, Central: Sep 29, 2014 Site, Local: /
Russia	Independent Interdisciplinary Committee for Ethics Expertise of Clinical Trials 51 Leningradsky prospect Moscow, 125468 Russia		RU90310 7 III International str. Ulyanovsk, Ul'yanovskaya oblast', 432018 Russian Federation Received drug	Region: Sep 29, 2014 Site, Central: Sep 29, 2014 Site, Local: /
Russia	Independent Interdisciplinary Committee for Ethics Expertise of Clinical Trials 51 Leningradsky prospect Moscow, 125468 Russia		RU90341 3 Pirogova Str Petrozavodsk, Karelia, 185019 Russia Did not receive drug	Region: Sep 29, 2014 Site, Central: Sep 29, 2014 Site, Local: /
Spain	FUNDACION PARA LA INVESTIGACION Hospital Universitario La Fe Avinguda Abril Martorell 106 Valencia, 46026 Spain		ES00516 Av. Generalitat Valenciana 50 Manises, Valencia Spain Received drug	Region: Nov 07, 2014 Site, Central: / Site, Local: Nov 07, 2014
Spain	EC OF HOSPITAL ARNAU DE VILANOVA Secretaría Del Ceic Calle San Clemente 12 CEIC N/A Valencia N/a, 46015 Spain		ES00521 C/ San Clemente s/n Valencia, 46012 Spain Received drug	Region: Nov 07, 2014 Site, Central: / Site, Local: Nov 07, 2014
Spain	CEIC EUSKADI C/ Donostia San Sebastian 1 DIRECCION DE FARMACIA DEL DEPARTEMENTO DE SANIDAD Vitoria, 01010 Spain		ES00523 Plaza De Cruces S/n Baracaldo, 48903 Spain Received drug	Region: Nov 07, 2014 Site, Central: / Site, Local: Nov 07, 2014
Spain	HOSPITAL DE CRUCES Plaza De Cruces Barakaldo, Vizacaya, 48903 Spain		ES00523 Plaza De Cruces S/n Baracaldo, 48903 Spain Received drug	Region: Nov 07, 2014 Site, Central: / Site, Local: Nov 07, 2014
Spain	EC OF HOSP. DE MÓSTOLES Secretaría Del Comité Ético Calle Rio Jucar S/n CEIC AREA 8 N/A Madrid, 28935 Spain		ES00529 C/ Gladiolo s/n Mostoles, 28933 Spain Did not receive drug	Region: Nov 07, 2014 Site, Central: / Site, Local: Nov 07, 2014
Spain	CEIC Illes Balears Conselleria De Salut I Consum C/ Cecili Matel, 18 Palma De Mallorca Mallorca, 07003 Spain		ES00536 Ctra. De Manacor, km 4 Son Ferriol, Baleares, 07198 Spain Received drug	Region: Nov 07, 2014 Site, Central: / Site, Local: Nov 07, 2014
Spain	CEIC Hospital Universitario Salamanca Paseo de San Vicente, 58-182 Salamanca, Salamanca, 37007 Spain		ES00538 Paseo de San Vicente 58-182 SALAMANCA, N/A, 37007 Spain Received drug	Region: Nov 07, 2014 Site, Central: / Site, Local: Nov 07, 2014
Spain	COMITE AUTONOMICO DE ENSAYOS CLINICOS DE ANDALUCIA Avda De La Innovacion S/n Edificio Arena 1 CONSEJERIA DE SALUD - SECRETARIA GENERAL DE CALIDAD Y MODERNIZACION Sevilla, 41071 Spain		ES00541 Avenida Menendez Pidal S/n Cordoba, 14004 Spain Received drug	Region: Nov 07, 2014 Site, Central: / Site, Local: Nov 07, 2014
Spain	EC OF Hospital Reina Sofia Avda Menendez Pidal s/n Cordoba, 14004 Spain		ES00541 Avenida Menendez Pidal S/n Cordoba, 14004 Spain Received drug	Region: Nov 07, 2014 Site, Central: / Site, Local: Nov 07, 2014
Spain	EC OF HOSPITAL UNIVERSITARIO DE GETAFE Carretera Madrid-Toledo Kilometro 12 500 N/A Madrid, 28905 Spain		ES00542 Mateo Inurria, s/n MADRID, MADRID, 28850 Spain Received drug	Region: Nov 07, 2014 Site, Central: / Site, Local: Nov 07, 2014
Spain	COMITE ETICO DE INVESTIGACION CLINICA DEL HOSPITAL UNIVERSITARI VALL D'HEBRON - 2A PLANTA PASEO VALL D'HEBRON, 119-129 INSTITUT DE RECERCA HOSPITAL UNIVERSITARI VALL D'HEBRON BARCELONA, 08035 Spain	ETHIC COMMITTEE SUPPORT UNIT Tomas Mireia	ES00543 Paseo De Vall D Hebrón S/n Barcelona N/a, 00000 Spain Received drug	Region: Nov 07, 2014 Site, Central: / Site, Local: Nov 07, 2014
Spain	CEIC HOSPITAL UNIVERSITARIO LA PAZ PASEO DE LA CASTELLANA, 261 MADRID, Madrid, 28046 Spain		ES00544 Paseo de Europa, 34 San Sebastian de los Reyes, Madrid, 28702 Spain Received drug	Region: Nov 07, 2014 Site, Central: / Site, Local: Nov 07, 2014
Taiwan	National Taiwan University Hospital REC No.1, Changde St., Zhongzheng Dist. Taipei, 10002 Taiwan		TW00035 7, Chung-Shan South Road, Taipei, 10002 Taiwan Received drug	Region: Oct 06, 2014 Site, Central: / Site, Local: Nov 26, 2014
Taiwan	Chang Gung Medical Foundation Institutional Review Board B2., No.123, Dinghu Rd Guishan Township 333 Taoyuan County, 333 Taiwan		TW00061 123 Ta-Pei Rd Niao-Sung Hsiang Kaohsiung, 83342 Taiwan Received drug	Region: Oct 06, 2014 Site, Central: / Site, Local: Oct 06, 2014
Taiwan	Chang Gung Medical Foundation Institutional Review Board B2., No.123, Dinghu Rd Guishan Township 333 Taoyuan County, 333 Taiwan		TW00076 No.5, Fusing St. Taoyuan County, 333 Taiwan Received drug	Region: Oct 06, 2014 Site, Central: / Site, Local: Oct 06, 2014
Taiwan	National Cheng Kung University Hospital Institutional Review Board 6F, OPD building, No. 138, Sheng-Li Road Tainan Taiwan		TW00077 138 Sheng-Li Rd Tainan, 704 Taiwan Received drug	Region: Oct 06, 2014 Site, Central: / Site, Local: Nov 11, 2014
Taiwan	Chang Gung Medical Foundation Institutional Review Board B2., No.123, Dinghu Rd Guishan Township 333 Taoyuan County, 333 Taiwan		TW00078 No.199, Tunhwa North Road Taipei Taiwan Received drug	Region: Oct 06, 2014 Site, Central: / Site, Local: Oct 06, 2014
Taiwan	Chung Shan Medical University Hospital IRB No,110, Sec.1, Chien-Kuo N. Road Taichung, 402 Taiwan		TW90068 No. 110, Section 1, Jiànguó North Rd, Nan District Taichung, 40201 Taiwan Received drug	Region: Oct 06, 2014 Site, Central: / Site, Local: Dec 18, 2014
USA	Sterling Institutional Review Board 6300 Powers Ferry Rd. Atlanta, GA, 30339 USA	Saltzman Steven L., MD	US00494 601 Medical Tower Norfolk, VA, 23507 USA Received drug	Region: / Site, Central: / Site, Local: Oct 06, 2014
USA	Sterling Institutional Review Board 6300 Powers Ferry Road Suite 600-351 Atlanta, GA, 30339 USA	Saltzman Steven L., MD	US01499 104 Se 1St Ave Ocala, FL, 34471 USA Received drug	Region: / Site, Central: / Site, Local: Sep 19, 2014
USA	Sterling Institutional Review Board 6300 Powers Ferry Road Suite 600-351 Atlanta, GA, 30339 USA	Saltzman Steven L., MD	US01500 1100 W Central Rd Arlington Heights, IL, 60005 USA Received drug	Region: / Site, Central: / Site, Local: Sep 19, 2014
USA	Sterling Institutional Review Board 6300 Powers Ferry Road Suite 600-351 Atlanta, GA, 30339 USA	Saltzman Steven L., MD	US01505 11800 Atlantis Pl Alpharetta, GA, 30022 USA Received drug	Region: / Site, Central: / Site, Local: Sep 22, 2014
USA	Sterling Institutional Review Board 6300 Powers Ferry Road Suite 600-351 Atlanta, GA, 30339 USA	Saltzman Steven L., MD	US01507 1034 S. Brentwood Blvd Saint Louis, MO, 63117 USA Received drug	Region: / Site, Central: / Site, Local: Oct 23, 2014
USA	Sterling Institutional Review Board 6300 Powers Ferry Rd. Atlanta, GA, 30339 USA	Saltzman Steven L., MD	US01511 8930 S Sepulveda Blvd. Los Angeles, CA, 90045 USA Received drug	Region: / Site, Central: / Site, Local: Oct 06, 2014
USA	Sterling Institutional Review Board 6300 Powers Ferry Rd. Atlanta, GA, 30339 USA	Saltzman Steven L., MD	US01512 1203 Coal Ave SE Albuquerque, NM, 87106 USA Received drug	Region: / Site, Central: / Site, Local: Nov 24, 2014
USA	Sterling Institutional Review Board 6300 Powers Ferry Rd. Atlanta, GA, 30339 USA	Saltzman Steven L., MD	US01513 451 North Texas Avenue Webster, TX, 77598 USA Received drug	Region: / Site, Central: / Site, Local: Sep 04, 2014
USA	Sterling Institutional Review Board 6300 Powers Ferry Road Suite 600-351 Atlanta, GA, 30339 USA	Saltzman Steven L., MD	US02019 59 One Mile Rd Ext Ste G East Windsor, NJ, 08520 USA Received drug	Region: / Site, Central: / Site, Local: Sep 30, 2014
USA	Sterling Institutional Review Board 6300 Powers Ferry Rd. Atlanta, GA, 30339 USA	Saltzman Steven L., MD	US02020 4499 Medical Dr San Antonio, TX, 78229 USA Received drug	Region: / Site, Central: / Site, Local: Dec 17, 2014
USA	Sterling Institutional Review Board 6300 Powers Ferry Rd. Atlanta, GA, 30339 USA	Saltzman Steven	US02021 711 E Lamar Blvd Arlington, TX, 76011 USA Received drug	Region: / Site, Central: / Site, Local: Nov 04, 2014
USA	Sterling Institutional Review Board 6300 Powers Ferry Rd. Atlanta, GA, 30339 USA	Saltzman Steven L., MD	US02022 2565 N.W. Lovejoy Portland, OR, 97210 USA Received drug	Region: / Site, Central: / Site, Local: Sep 19, 2014
USA	Sterling Institutional Review Board 6300 Powers Ferry Rd. Atlanta, GA, 30339 USA	Saltzman Steven L., MD	US02046 3900 Junius St Dallas, TX, 75246 USA Received drug	Region: / Site, Central: / Site, Local: Dec 10, 2014
USA	Colorado Multiple Institutional Review Board 13001 E. 17TH PLACE BUILDING 500, ROOM N3214 AURORA, CO, 80010 USA	Easterday Kenneth, Pharm.D.	US02051 13001 E. 17th Place Aurora, CO, 80045 USA Received drug	Region: / Site, Central: / Site, Local: Mar 02, 2015
USA	Sterling Institutional Review Board 6300 Powers Ferry Road Suite 600-351 Atlanta, GA, 30339 USA	Saltzman Steven L., MD	US02052 200 Lothrop Street Pittsburgh, PA, 25213 USA Received drug	Region: / Site, Central: / Site, Local: Nov 13, 2014
USA	Sterling Institutional Review Board 6300 Powers Ferry Road Suite 600-351 Atlanta, GA, 30339 USA	Saltzman Steven L., MD	US02074 9101 N Central Expy Dallas, TX, 75231 USA Received drug	Region: / Site, Central: / Site, Local: Sep 30, 2014
USA	Sterling Institutional Review Board 6300 Powers Ferry Road Suite 600-351 Atlanta, GA, 30339 USA	Saltzman Steven L., MD	US02111 5101 Commerce Drive Bakersfield, CA, 93309 USA Did not receive drug	Region: / Site, Central: / Site, Local: Oct 01, 2014
USA	Sterling Institutional Review Board 6300 Powers Ferry Road Suite 600-351 Atlanta, GA, 30339 USA	Saltzman Steven L., MD	US02139 3705 Medical Parkway Austin, TX, 78705 USA Received drug	Region: / Site, Central: / Site, Local: Oct 07, 2014
USA	Sterling Institutional Review Board 6300 Powers Ferry Road Suite 600-351 Atlanta, GA, 30339 USA	Saltzman Steven L., MD	US02140 2001 Santa Monica Blvd Santa Monica, CA, 90404 USA Did not receive drug	Region: / Site, Central: / Site, Local: Sep 26, 2014
USA	University of Connecticut Health Center IRB 263 Farmington Avenue Farmington, CT, 06030 USA	Hegde Upendra, M.D. Peterson Douglas	US02260 263 Farmington Ave Farmington, CT, 06030 USA Received drug	Region: / Site, Central: / Site, Local: Mar 15, 2015
USA	Sterling Institutional Review Board 6300 Powers Ferry Road Suite 600-351 Atlanta, GA, 30339 USA	Saltzman Steven L., MD	US02275 1524 Atwood Ave Johnston, RI, 02919 USA Received drug	Region: / Site, Central: / Site, Local: Nov 10, 2014
USA	Sterling Institutional Review Board 6300 Powers Ferry Rd. Atlanta, GA, 30339 USA	Saltzman Steven L., MD	US90232 255 Kirts Blvd Troy, MI, 48084 USA Received drug	Region: / Site, Central: / Site, Local: Oct 10, 2014
USA	Sterling Institutional Review Board 6300 Powers Ferry Rd. Atlanta, GA, 30339 USA	Saltzman Steven L., MD	US90807 7205 University Ave Ne Fridley, MN, 55432 USA Received drug	Region: / Site, Central: / Site, Local: Oct 10, 2014
USA	STERLING IRB 6319 Powers Ferry Rd Nw Ste 600-35 Atlanta, GA, 30339 USA	Saltzman Steven	US91488 9495 Sw Locust Street Portland, OR, 97223 USA Received drug	Region: / Site, Central: / Site, Local: Oct 08, 2014
USA	Sterling Institutional Review Board 6300 Powers Ferry Rd. Atlanta, GA, 30339 USA	Saltzman Steven L., MD	US91491 1201 S. Myrtle Ave. Clearwater, FL, 33757 USA Received drug	Region: / Site, Central: / Site, Local: Oct 13, 2014
USA	Partners Human Research Committee Massachusetts Genereal Hospital Boston, MA, 02114 USA	Gray Rosalyn	US91513 50 Staniford Boston, MA, 02114 USA Received drug	Region: / Site, Central: / Site, Local: Apr 01, 2015
USA	Sterling Institutional Review Board 6300 Powers Ferry Rd. Atlanta, GA, 30339 USA	Saltzman Steven L., MD	US91723 501 Washington St San Diego, CA, 92103 USA Received drug	Region: / Site, Central: / Site, Local: Oct 23, 2014
USA	Sterling Institutional Review Board 6300 Powers Ferry Road Suite 600-351 Atlanta, GA, 30339 USA	Saltzman Steven L., MD	US92606 1100 Southfield Dr Plainfield, IN, 46168 USA Received drug	Region: / Site, Central: / Site, Local: Sep 19, 2014
USA	Sterling Institutional Review Board 6300 Powers Ferry Rd. Atlanta, GA, 30339 USA	Saltzman Steven L., MD	US93365 836 Ponce De Leon Blvd Coral Gables, FL, 33134 USA Received drug	Region: / Site, Central: / Site, Local: Oct 16, 2014
USA	SANFRANCISCO GENERAL HOSPITAL 995 Potrero Avenue N/A N/A San Francisco, CA, 94110 USA	Bersot Thomas P.	US93367 185 Berry Street San Francisco, CA, 94107 USA Received drug	Region: / Site, Central: / Site, Local: Nov 13, 2014
